# Luteolin Modulates Neural Stem Cells Fate Determination: *In vitro* Study on Human Neural Stem Cells, and *in vivo* Study on LPS-Induced Depression Mice Model

**DOI:** 10.3389/fcell.2021.753279

**Published:** 2021-11-01

**Authors:** Mariem Achour, Farhana Ferdousi, Kazunori Sasaki, Hiroko Isoda

**Affiliations:** ^1^Laboratory of Metabolic Biophysics and Applied Pharmacology, Faculty of Medicine of Sousse, University of Sousse, Sousse, Tunisia; ^2^Alliance for Research on the Mediterranean and North Africa (ARENA), University of Tsukuba, Tsukuba, Japan; ^3^Faculty of Life and Environmental Sciences, University of Tsukuba, Tsukuba, Japan; ^4^National Institute of Advanced Industrial Science and Technology (AIST)-University of Tsukuba Open Innovation Laboratory for Food and Medicinal Resource Engineering (FoodMed-OIL), University of Tsukuba, Tsukuba, Japan

**Keywords:** luteolin, human neural stem cells, stem cell differentiation, astrogenesis, AICAR, LPS-induced depression model, NSCs isolation, astrocyte isolation

## Abstract

Luteolin is a natural flavone with neurotrophic effects observed on different neuronal cell lines. In the present study, we aimed to assess the effect of luteolin on hNSCs fate determination and the LPS-induced neuroinflammation in a mouse model of depression with astrocytogenesis defect. hNSCs were cultured in basal cell culture medium (control) or medium supplemented with luteolin or AICAR, a known inducer of astrogenesis. A whole-genome transcriptomic analysis showed that luteolin upregulated the expressions of genes related to neurotrophin, dopaminergic, hippo, and Wnt signaling pathways, and downregulated the genes involved in p53, TNF, FOXO, and Notch signaling pathways. We also found that astrocyte-specific gene GFAP, as well as other genes of the key signaling pathways involved in astrogenesis such as Wnt, BMP, and JAK-STAT pathways were upregulated in luteolin-treated hNSCs. On the other hand, neurogenesis and oligodendrogenesis-related genes, *TUBB3*, *NEUROD 1 and 6*, and *MBP*, were downregulated in luteolin-treated hNSCs. Furthermore, immunostaining showed that percentages of GFAP+ cells were significantly higher in luteolin- and AICAR-treated hNSCs compared to control hNSCs. Additionally, RT-qPCR results showed that luteolin upregulated the expressions of *GFAP*, *BMP2*, and *STAT3*, whereas the expression of *TUBB3* remained unchanged. Next, we evaluated the effects of luteolin in LPS-induced mice model of depression that represents defects in astrocytogenesis. We found that oral administration of luteolin (10 mg/Kg) for eight consecutive days could decrease the immobility time on tail suspension test, a mouse behavioral test measuring depression-like behavior, and attenuate LPS-induced inflammatory responses by significantly decreasing IL-6 production in mice brain-derived astrocytes and serum, and TNFα and corticosterone levels in serum. Luteolin treatment also significantly increased mature BDNF, dopamine, and noradrenaline levels in the hypothalamus of LPS-induced depression mice. Though the behavioral effects of luteolin did not reach statistical significance, global gene expression analyses of mice hippocampus and brain-derived NSCs highlighted the modulatory effects of luteolin on different signaling pathways involved in the pathophysiology of depression. Altogether, our findings suggest an astrocytogenic potential of luteolin and its possible therapeutic benefits in neuroinflammatory and neurodegenerative diseases. However, further studies are required to identify the specific mechanism of action of luteolin.

## Introduction

Neural stem cells (NSCs) are self-renewal cells that can be differentiated into neurons or glial cells following neurogenesis and gliogenesis processes, respectively (Apple et al., 2017). For most of the 20th century, it was believed that neurogenesis is restricted to a limited period during development and ceased shortly after birth (Cajal, 1930; Ming and Song, 2011; Taverna et al., 2014; Apple et al., 2017). However, Eriksson et al., have shown that neurogenesis is a life-long continuous process in almost all mammals, including humans (Eriksson et al., 1998). Peng et al. have determined that adult neurogenesis occurs in three different regions of human and mouse brain, namely the subventricular zone, the striatum and the hippocampus (Peng and Bonaguidi, 2018). Recent evidence pointed out that the neurogenesis process is both behaviorally and biochemically linked to different psychiatric and neurodegenerative diseases (Apple et al., 2017; Peng and Bonaguidi, 2018), while other evidence highlighted the crucial roles of astrocytes and astrogenesis in the installation of central nervous system (CNS) pathologies, suggesting that the re-establishment or the enhancing of normal astrocytic functions may be of great therapeutic interest (Lee et al., 2015). Astrocytes, the largest and the most prevalent glial cell type in CNS, maintain the homeostasis control of the blood-brain barrier, and dynamically modulate synapse formation, maturation, and plasticity processes (Sahay et al., 2011; Akers et al., 2014). Defects in astrogenesis or early functions of astrocytes are reported to be involved in the development of different psychiatric disorders (Gonzales et al., 2017; Cohen and Torres, 2019; Pajarillo et al., 2019; Valles et al., 2019). Therefore, both neurons and astrocytes should be targeted simultaneously to re-establish the physiological functions in damaged brain. Although NSCs proliferation and differentiation processes are spontaneously stimulated in pathological conditions, they cannot overcome the damage and restore the brain tissue. It is in this context that pharmacological stimulation of NSCs fate determination plays an important role in cell therapy and regenerative medicine in debilitating brain diseases (Zhuang et al., 2012). Numerous studies have shown that small molecules, such as growth factors and retinoic acid, can regulate the biological characteristics of neural stem cells and promote neurogenesis and astrocytogenesis (Jacobs et al., 2006; Leker et al., 2009; Lee et al., 2015). However, the increasing incidences of lack of efficacy and undesirable side effects of existing pharmacological intervention have led to particular attention to several medicinal plants and their bioactive compounds. In this context, different studies have reported the ability of phytochemicals to target NSCs for inducing brain self-repair through modulating neurogenesis (Matias et al., 2016; Gonzales et al., 2017; Sasaki et al., 2019).

The natural flavone luteolin (3′, 4′, 5, 7-tetrahydroxyflavone), present in several edible plants, in fruits and tea including rosemary tea (Miean and Mohamed, 2001; Achour et al., 2018; Aziz et al., 2018), has been reported to exert anti-inflammatory, anti-carcinogenic, cardioprotective effects as well as several neurotrophic benefits (Luo et al., 2017; Aziz et al., 2018; Imran et al., 2019). It enhances the cholinergic activity, increases the expressions of neuronal differentiation markers and induces the neurites outgrowth in PC12 cells, a neuronal cell model derived from a pheochromocytoma of rat adrenal medulla and in SH-SY5Y cells, a serotoninergic neuronal cell model (Lin et al., 2010; El Omri et al., 2012; Bandaruk et al., 2014). Besides, the neuroprotective effects of luteolin have been reported in Alzheimer’s disease models and were explained by its antioxidant and anti-inflammatory properties (Sawmiller et al., 2014; Wang et al., 2016a). Despite all the aforementioned promising beneficial effects of luteolin on neural cells and neurodegenerative disease models, its effects on human neural stem cells (hNSCs) have never been explored.

Recently, neuro-inflammation was shown to be involved in the pathogenesis of different CNS diseases such as depression (Wu et al., 2019). In addition, it was reported that defects occurred in astrocytes and neurons are associated with this neuroinflammatory pathology and that natural compounds may exert an antidepressant effect by rewarding this defect (Lee and Giuliani, 2019; Wu et al., 2019).

The present study aimed to assess the effect of luteolin on hNSCs fate determination and to explore the effect of luteolin on a pathological animal model with astrocytogenesis defect, the LPS-induced depression mice.

## Materials and Methods

### Treatment Solutions Preparation

Luteolin, C_15_H_10_O_6_ (Supplementary Figure 1A) was purchased from Sigma-Aldrich Co., Ltd. (St. Louis, MO, United States). To prepare the stock solution, luteolin was dissolved in Dimethyl sulfoxide (DMSO). For *in vitro* assays, luteolin stock was dissolved in the cell culture medium and for animal experiment it was dissolved in PBS.

AICAR, C_9_H_15_N_4_O_8_ (Supplementary Figure 1B), is an adenosine analog and extensively used to activate 5′ adenosine monophosphate (AMP)-activated protein kinase (AMPK). This molecule was used in the present study since it is reported to induce the differentiation of rodent neural stem cells into astrocytes (Zang et al., 2008). It was purchased from Sigma-Aldrich Co., Ltd. (St. Louis, MO, United States). To prepare the stock solution, AICAR was dissolved in Dimethyl sulfoxide (DMSO), and then, for *in vitro* assays, AICAR stock was dissolved in the cell culture medium.

The Lipopolysaccharide (LPS), is the major component of the outer membrane of Gram-negative bacteria. It was used in the present study to induce depression in mice. This molecule was purchased from Wako (Japan). To prepare the mice treatment solution, LPS was dissolved in PBS.

### SH-SY5Y Cells Culture

The human neurotypic SH-SY5Y cells were purchased from the American Type Culture Collection (ATCC). The cells were cultured in 100 mm petri dish or 96-well plates depending on the purpose, with a 1:1 (v/v) mixture of Dulbecco’s minimum essential medium (Sigma, United States) and Ham’s F-12 nutrient mixture (Sigma, United States) supplemented with 15% fetal bovine serum (Sigma, United States), and 1% penicillin (5,000 μg/mL)-streptomycin (5,000 IU/mL) solution (ICN Biomedicals, Japan) and 1% non-essential amino-acid at 37°C in a 95% humidified air-5% CO2 incubator.

### Experiment on Human Neural Stem Cells

#### Human Neural Stem Cells Culture

The hNSCs (StemPro^TM^ Neural Stem Cells) are cryopreserved human fetal brain-derived neural stem cells derived from cortex of a male fetus donor aged 16 weeks that were purchased from Gibco, United States (Cat. no. A15654). It was cultured in T25 flasks (BD Falcon), or in 6-well plates (BD Falcon) with KnockOut^TM^ DMEM/F-12 (Gibco, United States, cat. no. 12660012) supplemented with 2% StemPro^TM^ Neural Supplement (Gibco, Cat. no. A1050801), 20 ng/mL of fibroblast growth factor (FGF) basic recombinant human, 20 ng/mL epidermal growth factor (EGF) recombinant human and 2 mM GlutaMAX^TM^-I Supplement (Cat. no. 35050), 6 U/mL heparin (Sigma, Cat. no. H3149), and 200 μM ascorbic acid (Sigma, Cat. no. A8960). For adhesion, Geltrex^®^ (Gibco, Cat. no. A14133) was used to coat the 6-well plate. The hNSCs were cultured at 37°C in a 95% air/5% CO2 humidified incubator. The medium was changed every 2 days.

#### Determination of Human Neural Stem Cells Viability

Cell viability was determined using a tetrazolium salt reduction assay: the MTT (3-[4,5-dimethylthiazol-2-yl]-2,5-diphenyltetrazo-lium bromide) assay on the human neurotypic SH-SY5Y cells. Cells were seeded at the density of 2 × 10^5^ on 96 well plates and treated with 1–50 μM of luteolin or 0.5–500 μM of AICAR for 48 h. Treatment solutions were made by dilution of luteolin and AICAR solutions in low reduced-serum minimal essential medium (OptiMEM^TM^, Gibco). After treatment, 5 mg/ml of MTT was added, and the cells were incubated for further 12 h. The MTT formazan was dissolved in 100 μl of 10% SDS (w/v). Absorbance was measured at 570 nm using a microtiter plate reader after overnight incubation (Biotech, United States).

#### Human Neural Stem Cells Differentiation Assay

Differentiation of hNSCs was induced following the previously described protocol (Sasaki et al., 2019). Briefly, hNSCs were seeded into 6 wells culture vessels coated with Geltrex^®^ at a density of 2.5 × 10^4^ cells/cm^2^. After 48 h, growth medium was replaced by differentiation medium: Knockout DMEM/F12 supplemented with 2% StemPro^®^ neural supplement, 2 mM GlutaMAX-I supplement, 6 units/mL heparin and 200 μM ascorbic acid, supplemented or not with 1 μM of Luteolin or 1 μM of AICAR. Throughout the study, differenciated hNSCs were used at P_0_.

#### Immunocytochemistry on Human Neural Stem Cells

For immunocytochemistry, cells were seeded in the Nunc Lab-Tek Chamber Slide System (Thermo Scientific, Japan) coated with Geltrex^®^ at the density of 2.5 × 10^4^ cells/cm^2^ and were incubated at 37°C, 5% CO_2_ for 48 h. After incubation, cells were treated with a differentiation medium supplemented or not 1 μM luteolin or 1 μM AICAR at 37°C for 24 h. After incubation, the cells were washed twice with PBS and fixed using 4% (w/v) cold formaldehyde (Wako, Japan) diluted in cold PBS for 30 min at room temperature. The fixed cells were later washed three times with PBS and permeabilized using 0.2% Triton-X surfactant (Sigma Aldrich, United States) diluted in PBS for 5 min at room temperature. After removing the Triton-X solution, cells were washed twice again using PBS, then blocked for 1 h at room temperature by 5% Fetal Goat Serum (Sigma, Japan) diluted in PBS. After incubation, the blocking buffer was removed and replaced by the diluted primary antibody solution. We used the primary antibodies of the rabbit polyclonal anti-Glial fibrillary acidic protein (GFAP) primary antibody (Cat. no. ab7260), the mouse monoclonal anti-beta III Tubulin (β3-tubulin) primary antibody (Cat. no. ab78078) and the rabbit polyclonal anti-Myelin Basic Protein (MBP) primary antibody (Cat. no. ab124493) to stain astrocytes, neurons, and oligodendrocytes, respectively. The antibodies were purchased from Abcam (Japan) and were diluted in 1% Fetal Goat Serum in PBS before use. After overnight incubation with primary antibodies at 4°C, cells were washed with PBS and incubated with the secondary antibodies solution Goat-anti mouse Alexa Fluor^®^ 594 (ab150116, Abcam, Japan) and Goat-anti-rabbit IgG H&L Alexa Fluor^®^ 488 (ab150077, Abcam, Japan) appropriately diluted in 1% Fetal Goat Serum in PBS for 2 h at room temperature avoiding light exposure. After incubation, cells were washed with PBS, and nuclei were counterstained with DAPI using drops of ProLong Diamond Antifade Mountant (Thermo Scientific, Japan). Fluorescence was detected with the IXplore Pro microscope system (OLYMPUS CORPORATION, Japan) monitored by OLYMPUS CellSens Dimension 1.18 software version XV3.17 (OLYMPUS CORPORATION, Japan, Copyright 2009–2017). Automated multichannel Cell count and cell imaging were performed by CellSens Dimension 1.18 software at magnification ×10.

### Animal Experiments

#### Animals, Treatment Protocol and Behavioral Test

Healthy adult male ICR mice (21 weeks old, weighting between 33 and 46 g) were used for animal experiments. All animals were purchased from Charles River Laboratories JAPAN Inc., Kanagawa, Japan and were housed individually at controlled temperature (25°C), with a 12/12 h light/dark cycle, and had access to food and water *ad libitum*. After acclimatization, the mice were randomly assigned into four experimental groups (*n* = 6/group): Control group (PBS), the depression model group: lipopolysaccharide group (LPS), control group treated with Luteolin (PBS + L) and lipopolysaccharide group treated with Luteolin (LPS + L). All experimental animals were maintained in accordance with the Guide for the Care and Use of Laboratory Animals, and the protocol was approved by the Animal Ethics Committee of the University of Tsukuba, Japan. At day 1 and prior to depression induction by LPS, the behavioral test: Tail Suspension Test (TST) was performed to all mice to screen their initial stress status. The TST methodology used in our study was as described by Steru (Steru et al., 1985). Briefly, the duration of the test was 6 min and the immobility time was measured on the last 4 min of the test. The mouse was considered immobile only when it is hanged passively, showing no resistance to the stress applied by the test. The experiment was recorded using a camera and scored by videos observations. Immediately after performing the TST test, LPS (850 μg/kg) was injected via the intraperitoneal route (i.p.) to the LPS and LPS + L groups and PBS was injected by the same route to the PBS and PBS + L groups. Starting from Day 2 and during eight consecutive days, luteolin (10 mg/kg) and PBS were orally administrated to mice once per day. Luteolin (10 mg/kg) was administrated to the LPS + L and PBS + L groups and PBS to both PBS and LPS groups. At day 9, a second TST test was conducted to evaluate the antidepressant effect exerted by luteolin on treated mice.

#### Brains Collection

After performing the second TST test on day 9, all mice were sacrificed by cervical dislocation. Two mice brains from each group were used to separate NSCs, two mice brains were used to separate astrocytes and the remaining two brains were washed twice with cold PBS and immersed in liquid nitrogen prior to their storage at −80°C.

#### Neural Stem Cells and Primary Astrocytes Separation

Prior to NSCs and astrocytes isolation, the whole mice brain tissues were dissociated using the Adult Brain Dissociation Kit (Miltenyi Biotec Inc., CA, United States, order no. 130-107-677) in accordance to the manufacturer’s instructions. This kit permitted to obtain single cell suspensions by combining mechanical dissociation with enzymatic degradation of the extracellular matrix. Briefly, the neural tissue was enzymatically digested using the kit components and the gentleMACS^TM^ Dissociators (Miltenyi Biotec Inc., CA, United States) were used for the mechanical dissociation steps. After dissociation, the Debris Removal Solution was used for the removal of debris followed by a subsequent removal of erythrocytes using the Red Blood Cell Removal Solution. Cells were processed immediately for separation.

Immediately after the brains tissues dissociations, the NSCs were separated using the Anti-Prominin-1 MicroBeads (Miltenyi Biotec Inc., CA, United States, order no. 130-092-333) and the astrocytes were isolated using the Anti-ACSA-2 (astrocyte cell surface antigen-2) MicroBeads kit (Miltenyi Biotec Inc., CA, United States, order no. 130-092-333) according the manufacturer’s instructions. Briefly, for the separation of NSCs, first the prominin-1 + cells were magnetically labeled with AntiProminin-1 MicroBeads. Then, the cell suspension was loaded onto a MACS^®^ Column, which was placed in the magnetic field of a MACS Separator. The magnetically labeled prominin-1 + cells were retained within the column. The unlabeled cells run through; this cell fraction is thus depleted of prominin-1 + cells. After removing the column from the magnetic field, the magnetically retained prominin-1 + cells were eluted as the positively selected cell fraction. To separate the astrocytes, first Fc receptors were blocked with the mouse FcR Blocking Reagent. Then, the ACSA-2 + cells were magnetically labeled with Anti-ACSA-2 MicroBeads. The cell suspension was loaded onto a MACS^®^ Column, which was placed in the magnetic field of a MACS Separator. The magnetically labeled ACSA-2 + cells were retained within the column. The unlabeled cells run through; this cell fraction was thus depleted of ACSA-2 + cells. After removing the column from the magnetic field, the magnetically retained ACSA-2 + cells were eluted as the positively selected cell fraction.

#### Count and Culture of Isolated Mice Primary Astrocytes

After separation with the Anti-ACSA-2 MicroBeads kit (Miltenyi Biotec Inc., CA, United States, order no. 130-092-333), the negative and positive ACSA-2 cells fractions were collected separately and counted using the Guava ViaCount Software (Version Number – 2.5.2), then the astrocyte fraction was cultured in 30 mm petri dish in DMEM, high glucose cell culture medium supplemented with 10% inactivated fetal bovine serum (Sigma, United States), and 1% penicillin (5,000 μg/mL)-streptomycin (5,000 IU/mL) solution (ICN Biomedicals, Japan) at 37°C in a 95% humidified air-5% CO2 incubator for 7 days.

#### ELISA Analysis

ELISA tests were performed on astrocytes culture media, on mice sera and on mice hypothalamus and frontal cortex tissues to confirm the installation of depression in LPS treated mice, and to confirm the antidepressant effect of luteolin in this mice model.

The pro-inflammatory cytokine IL-6 was quantified in the mice primary astrocyte culture medium at day 1, day 3, and day 7 and in mice sera using the mouse Quantikine^®^ ELISA kit (cat. number. M6000B, R&D Systems, United States). The Tumor Necrosis Factor TNFα, and the corticosterone levels were quantified in all mice sera using the Quantikine^®^ ELISA kit cat. number MTA00B purchased from R&D Systems, United States. The Corticosterone ELISA kit (cat. number ADI-900-097) purchased from Enzo Life Sciences, NY, United States was used to quantify corticosterone levels in mice sera.

The serotonin (Sert), noradrenaline (NA), dopamine, pro-and mature-BDNF levels were quantified in mice hypothalamus and frontal cortex (2 mice from each group were used). First, we homogenized 20 mg of tissue in 1 mL of RIPA buffer. The homogenate was centrifuged for 5 min at 10,000 × *g*, 4°C. The supernatant was collected and stored at −80°C. The dopamine, Sert and NA were quantified using ELISA kits (Immusmol SAS, Talence, France). Pro and mature BDNF were measured by colorimetric sandwich ELISA kit (Proteintech, Rosemont, IL, United States). The experiments were conducted following the manufacturer’s instructions. The results of each treatment group were corrected by their respective total protein content determined using Pierce^TM^ BCA Protein Assay Kit (Cat. number 23225, Thermo Scientific, Japan).

### RNA Isolation

To extract hNSCs RNA, cells were seeded in 6 well plates at the density of 2.5 × 104 cells/cm2 and were incubated at 37°C for 48 h. After that, they were treated with differentiation medium with or without 1 μM of luteolin or 1 μM of AICAR and were incubated at 37°C for 8 and 24 h. After the treatment, cells were washed twice with ice-cold PBS (−). To extract RNA from mice hippocampus, two mice brains of each animal group were dissected and hippocampus tissues were used. The isolated NSCs from two mice brains were immediately used to extract total RNA.

The ISOGEN kit (Nippon Gene Co., Ltd., Japan) was use for all total RNA extraction experiments following the manufacturer’s instructions, as reported previously by Sasaki et al. (2019). Total RNA was purified using chloroform and Isopropanol (Wako, Japan) and quantified and assessed for quality with a NanoDrop 2000 spectrophotometer (Thermo Scientific, Wilmington, DE, United States).

### DNA Microarray Analysis

To elucidate the molecular mechanism underlying the effect of luteolin treatment on hNSCs fate determination, we compared the global gene expression of untreated and luteolin-treated hNSCs and to elucidate the molecular mechanism underlying the effect of luteolin treatment on LPS-induced depression model NSCs and hippocampus, we performed three global gene expression comparison studies. First, we compared the global gene expression of LPS group and control group (LPS vs. PBS), then we compared the global gene expression of control group treated with luteolin and untreated control group (PBS + L vs. PBS) and finally we compared the LPS group treated with luteolin and LPS group (LPS + L vs. LPS).

These genomic analyses were made by performing microarray analysis using Affymetrix GeneChip 3′ IVT PLUS reagent kit (Affymetrix Inc., Santa Clara, CA, United States) according to the kit user’s guide. Data normalization and transformation was done by Expression Console software. Subsequent analysis of the differentially expressed genes was performed using Transcriptome Analysis Console (version 4) and DAVID online tool (Huang et al., 2009; Sherman and Lempicki, 2009). Heatmap was generated using a freely available web tool Heatmapper (Babicki et al., 2016).

### Real-Time Polymerase Chain Reaction Analysis on Human Neural Stem Cells

RNA extracts obtained from hNSCs treated or not with 1 μM luteolin for 8 and 24 h were used to validate the microarray data. Reverse transcription was performed using the Superscript IV reverse transcriptase kit (Invitrogen, United States) following the manufacturer’s guidelines. A mixture of RNA samples (30 ng/μl) and Oligo d (T)_20_/dNTP were incubated for 5 min at 5°C, then placed in ice for 1 min. The Superscript^®^ IV reverse transcriptase solution was added. The mixture was vortexed and centrifuged, then TaqMan probes specific to, Glial Fibrillary Acidic Protein (*GFAP*) (Hs 00909233_m1), Bone Morphogenetic Protein 2 (*BMP2*) (Hs00154192_m1), Signal Transducer and Activator of Transcription (*STAT3*) (Hs00374280_m1), Tubulin, beta 3 class III (*TUBB3*) (Hs00801390_s1), *NOTCH 1* (Hs01062014_m1), *NOTCH 3* (Hs00166432_m1), Myelin Basic Protein (*MBP*) (Hs 00921945_m1) genes and master mix TaqMan were added to 100 ng/9 μL of obtained cDNA, and the mixture was introduced to 7500 Fast Real-time PCR (Applied Biosystems, United States) and the following conditions were applied: 50°C for 2 min, followed by 95°C for 10 min, and 40 cycles of 95°C for 15 s followed by 60°C for 1 min. Glyceraldehyde-3-Phosphate Dehydrogenase (*GAPDH*) gene (Hs02786624_g1 GAPDH) was used as an endogenous control. All primers were purchased from (Applied Biosystems, CA, United States), and all reactions were run in triplicates.

### Statistical Analysis

Statistical analysis was performed using SPSS software (Version 24; IBM, Armonk, NY, United States). A Student’s *t*-test was used when two groups were compared in MTT assay, astrocytes quantification and all ELISA tests. Statistical analysis of the results obtained in the immunocytochemistry and the real-time polymerase chain reaction (RT-qPCR) validation results were carried out using one-way analysis of variance (ANOVA) followed by Dunnett’s *post hoc* test. The difference in signal intensities between control and luteolin-treated hNSCs was tested using student’s *t*-test. Statistical analysis of TST results were carried out using paired *t*-test to compare the same groups results obtained between Day 1 and Day 8, and using One-way ANOVA test to compare different groups results at Day 1 and at day 8. Results are expressed as mean ± standard deviation unless otherwise indicated. The criterion of statistical significance was *p* < 0.05.

## Results

### Results Obtained From the *in vitro* Study

#### Cell Viability

Results from MTT assay indicate that 1 μM luteolin slightly increased cell viability of SH-SY5Y cells after 24 h and 48 h treatment (*p* < 0.001), concentrations ranged from 5 to 10 μM did not affect cell viability while higher concentrations induced cell toxicity in a dose dependent-manner after 24 and 48 h treatment (Supplementary Figure 2). Treatment with 0.5 μM of AICAR for 24 h could slightly increase SH-SY5Y cell viability (104.1% ± 1.9, *p* < 0.01). Concentrations ranged between 0.5 and 5 μM did not affect cell viability, while higher concentration significantly reduced the viable SH-SY5Y cell number to 70% (Data not shown).

#### Luteolin Affected Global Gene Expression During Human Neural Stem Cells Differentiation

We evaluated the effect of luteolin on the transcriptomic changes in hNSCs during differentiation at 24 h using DNA microarray. We found that treatment with 1 μM luteolin could significantly regulate the expressions of 5870 genes in hNSCs (−1.3 < Fold change < 1.3; *p* < 0.05), with upregulation of 2638 genes and downregulation of 3232 genes. Top 10 significantly up and downregulated genes and their functions are presented in Supplementary Tables 1, 2, respectively.

**FIGURE 1:**
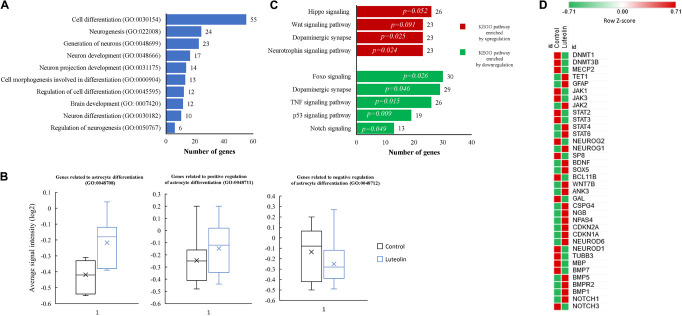
Luteolin affected biological processes and directed differentiation of hNSCs toward astrocytes. **(A)** Top 10 significantly enriched biological processes between 1 μM luteolin treated hNSCs and untreated control hNSCs. **(B)** Box plots showing the signal intensity of genes involved in astrocyte differentiation (7 genes averaged), positive regulation of astrocyte differentiation (13 genes averaged), and negative regulation of astrocyte differentiation (17 genes averaged). Each box represents the quartile values, the error bar represents the SD, the midline represents the median value and the multiplication sign denotes the mean in signal intensities between control and luteolin-treated hNSCs was tested using student’s *t*-test. **(C)** Top significantly regulated KEGG signaling pathways. **(D)** Heat map showing expression intensities (log2 transformed) of genes involved in NSC differentiation between luteolin-treated (1 μM) hNSCs and untreated control hNSCs (Heat map plotted using the following online tool http://www.heatmapper.ca/; Sasha Babicki, David Arndt, Ana Marcu, Yongjie Liang, Jason R. Grant, Adam Maciejewski, and David S. Wishart. Heatmapper: web-enabled heat mapping for all. Nucleic Acids Res. 2016 May 17).

Top 10 significantly enriched biological processes between 1 μM luteolin treated hNSCs and untreated control hNSCs are shown in Figure 1A. Fifty-five regulated genes were related to cell differentiation (GO:0030154), 24 to neurogenesis (GO:022008), 23 to generation of neurons (GO:0048699), 17 to neuron development (GO:0048666), 14 to neuron projection development (GO:0031175), 13 to cell morphogenesis involved in neurodifferentiation (GO:0048667), 12 to regulation of cell differentiation (GO: 0045595), 12 to brain development (GO:0007420), 10 to neuron differentiation, and 6 regulated genes to regulation of neurogenesis (GO:0050767). Besides, the essential gene expressions related to astrocyte differentiation biological process (GO:0048708), negative regulation of astrocyte differentiation (GO:0048712), and positive regulation of astrocyte differentiation (GO:0048712) were also regulated in luteolin treated hNSCs compared to untreated control cells (Figure 1B and Supplementary Tables 3–5).

Significantly regulated top KEGG signaling pathways by 1 μM luteolin treatment for 24 h are shown in Figure 1C. Luteolin treatment upregulated 26 genes in Hippo signaling (*p* = 0.052), 23 genes involved in Wnt signaling pathway (*p* = 0.091), 23 genes in the dopaminergic synapse (*p* = 0.025) and 23 genes involved in the Neurotrophin pathway (*p* = 0.024). Also, luteolin treatment downregulated 30 genes in FOXO signaling (*p* = 0.026), 29 genes in dopaminergic synapse (*p* = 0.046), 26 genes in TNF signaling (*p* = 0.015), 19 genes involved in p53 signaling pathway (*p* = 0.009), and 13 genes in notch signaling (*p* = 0.049) (Figure 1C and Table 1).

**TABLE 1:** Top significantly enriched KEGG signaling pathways in NSCs of LPS-induced depression mice (LPS) compared to untreated mice (PBS).

Regulation	KEGG signaling	Genes
Downregulation	**Estrogen signaling pathway (mmu04915)**	FBJ osteosarcoma oncogene **(*Fos*)**
		GNAS (guanine nucleotide binding protein, alpha stimulating) complex locus **(*Gnas)***
		cAMP responsive element binding protein 3 **(*Creb3*)**
		guanine nucleotide binding protein (G protein), alpha inhibiting 2 **(*Gnai2*)**
		heat shock protein 1B **(*Hspa1b*)**
		heat shock protein 8 **(*Hspa8*)**
		heat shock protein 90 alpha (cytosolic), class B member 1 **(*Hsp90ab1*)**
		heat shock protein 90, alpha (cytosolic), class A member 1 **(*Hsp90aa1*)**
		heat shock protein 90, beta (Grp94), member 1 **(*Hsp90b1*)**
		phosphatidylinositol 3-kinase catalytic delta polypeptide **(*Pik3cd*)**

	**Thyroid hormone synthesis (mmu04918)**	ATPase, Na+/K+ transporting, alpha 2 polypeptide **(*Atp1a2*)**
		ATPase, Na+/K+ transporting, beta 2 polypeptide **(*Atp1b2*)**
		GNAS (guanine nucleotide binding protein, alpha stimulating) complex locus **(*Gnas*)**
		cAMP responsive element binding protein 3 **(*Creb3*)**
		heat shock protein 90, beta (Grp94), member 1 **(*Hsp90b1*)**

	**Adherens junction (mmu04520)**	WAS protein family, member 2 **(*Wasf2*)**
		actin, beta **(*Actb*)**
		actin, gamma, cytoplasmic 1 **(*Actg1*)**
		catenin (cadherin associated protein), beta 1 **(*Ctnnb1*)**
		protein tyrosine phosphatase, receptor type, B **(*Ptprb*)**

	**cAMP signaling pathway (mmu04024)**	ATPase, Na+/K+ transporting, alpha 2 polypeptide **(*Atp1a2*)**
		ATPase, Na+/K+ transporting, beta 2 polypeptide **(*Atp1b2*)**
		FBJ osteosarcoma oncogene **(*Fos*)**
		GNAS (guanine nucleotide binding protein, alpha stimulating) complex locus **(*Gnas*)**
		cAMP responsive element binding protein 3 **(*Creb3)***
		calcium/calmodulin-dependent protein kinase II alpha **(*Camk2a*)**
		guanine nucleotide binding protein (G protein), alpha inhibiting 2 **(*Gnai2*)**
		nuclear factor of kappa light polypeptide gene enhancer in B cells inhibitor, alpha **(*Nfkbia*)**
		phosphatidylinositol 3-kinase catalytic delta polypeptide (*Pik3cd*)

	**Glutamatergic synapse (mmu04724)**	GNAS (guanine nucleotide binding protein, alpha stimulating) complex locus **(*Gnas*)**
		glutamate-ammonia ligase (glutamine synthetase) **(*Glul*)**
		guanine nucleotide binding protein (G protein), alpha inhibiting 2 **(*Gnai2*)**
		guanine nucleotide binding protein (G protein), beta 1 **(*Gnb1*)**
		predicted gene 15776 **(*Gm15776*)**
		solute carrier family 1 (glial high affinity glutamate transporter), member 2 **(*Slc1a2***)
		solute carrier family 1 (glial high affinity glutamate transporter), member 3 **(*Slc1a3*)**

	**Cholinergic synapse (mmu04725)**	FBJ osteosarcoma oncogene **(*Fos*)**
		cAMP responsive element binding protein 3 **(*Creb3*)**
		calcium/calmodulin-dependent protein kinase II alpha **(*Camk2a*)**
		guanine nucleotide binding protein (G protein), alpha inhibiting 2 **(*Gnai2*)**
		guanine nucleotide binding protein (G protein), beta 1 **(*Gnb1*)**
		phosphatidylinositol 3-kinase catalytic delta polypeptide **(*Pik3cd*)**
		predicted gene 15776 **(*Gm15776*)**

	**Dopaminergic synapse (mmu04728)**	FBJ osteosarcoma oncogene **(*Fos*)**
		GNAS (guanine nucleotide binding protein, alpha stimulating) complex locus (***Gnas***)
		cAMP responsive element binding protein 3 **(*Creb3*)**
		calcium/calmodulin-dependent protein kinase II alpha **(*Camk2a*)**
		guanine nucleotide binding protein (G protein), alpha inhibiting 2 **(*Gnai2*)**
		guanine nucleotide binding protein (G protein), beta 1 **(*Gnb1*)**
		predicted gene 15776 **(*Gm15776*)**
		protein phosphatase 2, regulatory subunit B, alpha **(*Ppp2r2a*)**

	**PI3K-Akt signaling pathway (mmu04151)**	FMS-like tyrosine kinase 1 **(*Flt1*)**
		Von Willebrand factor **(*Vwf*)**
		cAMP responsive element binding protein 3 **(*Creb3*)**
		eukaryotic translation initiation factor 4E member 2 **(*Eif4e2*)**
		fibronectin 1 **(*Fn1*)**
		guanine nucleotide binding protein (G protein), beta 1 **(*Gnb1*)**
		heat shock protein 90 alpha (cytosolic), class B member 1 **(*Hsp90ab1*)**
		heat shock protein 90, alpha (cytosolic), class A member 1 **(*Hsp90aa1*)**
		heat shock protein 90, beta (Grp94), member 1 **(*Hsp90b1*)**
		phosphatidylinositol 3-kinase catalytic delta polypeptide **(*Pik3cd*)**
		predicted gene 15776 **(*Gm15776*)**
		protein phosphatase 2, regulatory subunit B, alpha **(*Ppp2r2a*)**
		ribosomal protein S6 **(*Rps6*)**
		tyrosine 3-monooxygenase/tryptophan 5-monooxygenase activation protein, epsilon polypeptide **(*Ywhae*)**
		tyrosine 3-monooxygenase/tryptophan 5-monooxygenase activation protein, theta polypeptide **(*Ywhaq*)**

Downregulation	**Thyroid hormone signaling pathway (mmu04919)**	ATPase, Na+/K+ transporting, alpha 2 polypeptide (***Atp1a2***)
		ATPase, Na+/K+ transporting, beta 2 polypeptide (***Atp1b2***)
		actin, beta (***Actb***)
		actin, gamma, cytoplasmic 1 (***Actg1*)**
		catenin (cadherin associated protein), beta 1 (***Ctnnb1***)
		phosphatidylinositol 3-kinase catalytic delta polypeptide (***Pik3cd***)
		solute carrier family 2 (facilitated glucose transporter), member 1 (***Slc2a1***)
		solute carrier organic anion transporter family, member 1c1 (***Slco1c1***)

	**Gap junction (mmu04540)**	GNAS (guanine nucleotide binding protein, alpha stimulating) complex locus (***Gnas***)
		Gap junction protein, alpha 1 (***Gja1***)
		Gap junction protein, delta 2 (***Gjd2***)
		guanine nucleotide binding protein (G protein), alpha inhibiting 2 (***Gnai2***)
		tubulin, alpha 1A (***Tuba1a***)
		tubulin, alpha 1B (***Tuba1b***)

	**Insulin secretion (mmu04911)**	ATPase, Na+/K+ transporting, alpha 2 polypeptide (***Atp1a2***)
		ATPase, Na+/K+ transporting, beta 2 polypeptide (***Atp1b2***)
		GNAS (guanine nucleotide binding protein, alpha stimulating) complex locus (***Gnas***)
		RAB3A, member RAS oncogene family (***Rab3a***)
		cAMP responsive element binding protein 3 (***Creb3***)
		calcium/calmodulin-dependent protein kinase II alpha (***Camk2a***)
		solute carrier family 2 (facilitated glucose transporter), member 1 (***Slc2a1***)

Upregulation	**Salivary secretion (mmu04970)**	cathelicidin antimicrobial peptide (***Camp***)
		lysozyme 1 (***Lyz1***)
		lysozyme 2 (***Lyz2***)

	**Systemic lupus erythematosus (mmu05322)**	H2A histone family, member Z (***H2afz***)
		cathepsin G (***Ctsg***)
		histone cluster 1, H2ac (***Hist1h2ac***)

*Gene abbreviations are indicated in bold.*

We further evaluated the effect of 24 h treatment with 1 μM luteolin on 37 selected genes that were reported to be involved in neural stem cells (NSCs) differentiation process (Yao and Jin, 2014; Hsieh and Zhao, 2016; Figure 1D). We found that luteolin treatment upregulated the expressions of 17 genes and downregulated the expressions of 20 genes (−1.3 < Fold change < 1.3; *p* < 0.05; Figure 1D and Table 2). It downregulated the expressions of neurogenin 2 (*NEUROG2*), DNA (cytosine-5-)-methyl transferase 1 (*DNMT1*), DNA (cytosine-5-)-methyl transferase 3 beta (*DNMT3B*), neuronal differentiation 1 (*NEUROD*1), neuronal differentiation 6 (*NEUROD6)*, *NOTCH3*, bone morphogenic protein (*BMP7*), and *TUBB3* that are known to regulate neuron differentiation. The expression of methyl-CpG binding protein 2 (*MECP2*) that is known to regulate neuron maturation, was also downregulated (Figure 1D and Table 2). Besides, luteolin treatment regulated several genes related to astrocyte development, astrocyte differentiation, and signaling pathways that direct astrocytogenesis. Luteolin upregulated Wnt family member 7B (*WNT7B)*, *GFAP*, and Janus kinase (*JAK*) 2 expression (Figure 1D and Table 2), whereas downregulated the most important oligodendrocyte genesis gene, *MBP*.

**TABLE 2:** Top significantly enriched KEGG signaling pathways in NSCs of LPS-induced depression mice treated with luteolin (LPS + L) compared to untreated mice LPS-induced depression mice (LPS).

Regulation	KEGG signaling	Genes
Upregulation	**Parkinson’s disease (mmu05012)**	ATP synthase, H+ transporting, mitochondrial F0 complex, subunit C3 (subunit 9) (***Atp5g3***)
		ATP synthase, H+ transporting, mitochondrial F0 complex, subunit D **(*Atp5h*)**
		ATP synthase, H+ transporting, mitochondrial F1 complex, O subunit (***Atp5o*)**
		ATP synthase, H+ transporting, mitochondrial F1 complex, epsilon subunit **(*Atp5e*)**
		ATP synthase, H+ transporting, mitochondrial F1 complex, gamma polypeptide 1 **(*Atp5c1*)**
		NADH dehydrogenase (ubiquinone) 1 alpha subcomplex, 13 **(*Ndufa13*)**
		NADH dehydrogenase (ubiquinone) 1 alpha subcomplex, 3 **(*Ndufa3*)**
		NADH dehydrogenase (ubiquinone) 1 alpha subcomplex, 9 **(*Ndufa9*)**
		NADH dehydrogenase (ubiquinone) 1 beta subcomplex, 11 **(*Ndufb11*)**
		NADH dehydrogenase (ubiquinone) 1 beta subcomplex, 5 **(*Ndufb5*)**
		NADH dehydrogenase (ubiquinone) flavoprotein 3 **(*Ndufv3*)**
		NADH dehydrogenase [ubiquinone] 1 subunit C2 **(*LOC102641347*)**
		NADH dehydrogenase subunit 5 **(*ND5*)**
		cytochrome c oxidase subunit IV isoform 1 **(*Cox4i1*)**
		cytochrome c oxidase subunit VIIa 2 **(*Cox7a2*)**
		cytochrome c oxidase subunit VIIa polypeptide 2-like **(*Cox7a2l*)**
		cytochrome c oxidase subunit VIa polypeptide 1 **(*Cox6a1*)**
		cytochrome c oxidase subunit Va **(*Cox5a*)**
		cytochrome c oxidase subunit Vb **(*Cox5b*)**
		cytochrome c oxidase, subunit VIb polypeptide 1 **(*Cox6b1*)**
		guanine nucleotide binding protein (G protein), alpha inhibiting 2 **(*Gnai2*)**
		solute carrier family 25 (mitochondrial carrier, adenine nucleotide translocator), member 4 **(*Slc25a4*)**
		solute carrier family 25 (mitochondrial carrier, adenine nucleotide translocator), member 5 **(*Slc25a5*)**
		ubiquinol-cytochrome c reductase binding protein **(*Uqcrb*)**

	**Alzheimer’s disease (mmu05010)**	ATP synthase, H+ transporting, mitochondrial F0 complex, subunit D **(*Atp5h*)**
		ATP synthase, H+ transporting, mitochondrial F1 complex, O subunit **(*Atp5o*)**
		ATP synthase, H+ transporting, mitochondrial F1 complex, epsilon subunit *(**Atp5e**)*
		ATP synthase, H+ transporting, mitochondrial F1 complex, gamma polypeptide 1 (***Atp5c1***)
		ATPase, Ca++ transporting, cardiac muscle, slow twitch 2 (***Atp2a2***)
		ATP synthase, H+ transporting, mitochondrial F0 complex, subunit D (***Atp5h***)
		ATP synthase, H+ transporting, mitochondrial F1 complex, O subunit (***Atp5o***)
		NADH dehydrogenase (ubiquinone) 1 alpha subcomplex, 13 **(*Ndufa13*)**
		NADH dehydrogenase (ubiquinone) 1 alpha subcomplex, 3 **(*Ndufa3*)**
		NADH dehydrogenase (ubiquinone) 1 alpha subcomplex, 9 **(*Ndufa9*)**
		NADH dehydrogenase (ubiquinone) 1 beta subcomplex, 11 **(*Ndufb11*)**
		NADH dehydrogenase (ubiquinone) 1 beta subcomplex, 5 **(*Ndufb5*)**
		NADH dehydrogenase (ubiquinone) flavoprotein 3 **(*Ndufv3*)**
		NADH dehydrogenase [ubiquinone] 1 subunit C2 **(*LOC102641347*)**
		amyloid beta (A4) precursor protein **(*App*)**
		apolipoprotein E **(*Apoe*)**
		calmodulin 1 **(*Calm1*)**
		cytochrome c oxidase subunit IV isoform 1 **(*Cox4i1*)**
		cytochrome c oxidase subunit VIIa 2 **(*Cox7a2*)**
		cytochrome c oxidase subunit VIIa polypeptide 2-like **(*Cox7a2l*)**
		cytochrome c oxidase subunit VIa polypeptide 1 **(*Cox6a1*)**
		cytochrome c oxidase subunit Va **(*Cox5a*)**
		cytochrome c oxidase subunit Vb **(*Cox5b*)**
		cytochrome c oxidase, subunit VIb polypeptide 1 **(*Cox6b1*)**
		glyceraldehyde-3-phosphate dehydrogenase, pseudogene 15 **(*Gapdh-ps15*)**
		presenilin enhancer gamma secretase subunit **(*Psenen*)**
		ubiquinol-cytochrome c reductase binding protein **(*Uqcrb*)**

	**Huntington’s disease (mmu05016)**	ATP synthase, H+ transporting, mitochondrial F0 complex, subunit C3 (subunit 9) **(*Atp5g3*)**
		ATP synthase, H+ transporting, mitochondrial F0 complex, subunit D **(*Atp5h*)**
		ATP synthase, H+ transporting, mitochondrial F1 complex, O subunit (***Atp5o***)
		ATP synthase, H+ transporting, mitochondrial F1 complex, epsilon subunit (***Atp5e***)
		ATP synthase, H+ transporting, mitochondrial F1 complex, gamma polypeptide 1 (***Atp5c1***)
		NADH dehydrogenase (ubiquinone) 1 alpha subcomplex, 13 (***Ndufa13***)
		NADH dehydrogenase (ubiquinone) 1 alpha subcomplex, 3 (***Ndufa3***)
		NADH dehydrogenase (ubiquinone) 1 alpha subcomplex, 9 (***Ndufa9***)
		NADH dehydrogenase (ubiquinone) 1 beta subcomplex, 11 (***Ndufb11***)
		NADH dehydrogenase (ubiquinone) 1 beta subcomplex, 5 (***Ndufb5***)
		NADH dehydrogenase (ubiquinone) flavoprotein 3 (***Ndufv3***)
		NADH dehydrogenase [ubiquinone] 1 subunit C2 (***LOC102641347***)
		cAMP responsive element binding protein 3 (***Creb3***)
		clathrin, light polypeptide (Lca) (***Clta***)
		cytochrome c oxidase subunit IV isoform 1 (***Cox4i1***)
		cytochrome c oxidase subunit VIIa 2 (***Cox7a2***)
		cytochrome c oxidase subunit VIIa polypeptide 2-like (***Cox7a2l***)
		cytochrome c oxidase subunit VIa polypeptide 1 (***Cox6a1***)
		cytochrome c oxidase subunit Va (***Cox5a***)
		cytochrome c oxidase subunit Vb (***Cox5b***)
		cytochrome c oxidase, subunit VIb polypeptide 1 (***Cox6b1***)
		dynactin 1 (***Dctn1***)
		solute carrier family 25 (mitochondrial carrier, adenine nucleotide translocator), member 4 (***Slc25a4***)
		solute carrier family 25 (mitochondrial carrier, adenine nucleotide translocator), member 5 (***Slc25a5***)
		superoxide dismutase 1, soluble (***Sod1***)
		ubiquinol-cytochrome c reductase binding protein (***Uqcrb***)

Upregulation	**Estrogen signaling pathway (mmu04915)**	GNAS (guanine nucleotide binding protein, alpha stimulating) complex locus (***Gnas***)
		cAMP responsive element binding protein 3 (***Creb3)***
		calmodulin 1 (***Calm1***)
		guanine nucleotide binding protein (G protein), alpha inhibiting 2 (***Gnai2***)
		heat shock protein 1B (***Hspa1b***)
		heat shock protein 8 (***Hspa8***)
		heat shock protein 90 alpha (cytosolic), class B member 1 (***Hsp90ab1***)
		heat shock protein 90, alpha (cytosolic), class A member 1 (***Hsp90aa1***)
		heat shock protein 90, beta (Grp94), member 1 (***Hsp90b1***)
		phosphatidylinositol 3-kinase catalytic delta polypeptide (***Pik3cd***)

	**Thyroid hormone signaling pathway (mmu4919)**	ATPase, Na+/K+ transporting, alpha 2 polypeptide (***Atp1a2***)
		ATPase, Na+/K+ transporting, beta 2 polypeptide (***Atp1b2***)
		actin, beta (***Actb***)
		actin, gamma, cytoplasmic 1 (***Actg1***)
		catenin (cadherin associated protein), beta 1 (***Ctnnb1***)
		phosphatidylinositol 3-kinase catalytic delta polypeptide (***Pik3cd***)
		phospholipase C, gamma 1 (***Plcg1***)
		solute carrier family 16 (monocarboxylic acid transporters), member 2 (***Slc16a2***)
		solute carrier family 2 (facilitated glucose transporter), member 1 (***Slc2a1***)
		solute carrier organic anion transporter family, member 1c1 (***Slco1c1***)

	**HIF-1 signaling pathway (04066)**	FMS-like tyrosine kinase 1 (***Flt1***)
		Enolase 1, alpha non-neuron (***Eno1***)
		Glyceraldehyde-3-phosphate dehydrogenase, pseudogene 15 (***Gapdh-ps15***)
		phosphatidylinositol 3-kinase catalytic delta polypeptide (***Pik3cd***)
		phospholipase C, gamma 1 (***Plcg1***)
		predicted gene 9840 (***Gm9840***)
		ribosomal protein S6 (***Rps6***)
		solute carrier family 2 (facilitated glucose transporter), member 1 (***Slc2a1***)
		transferrin receptor (***Tfrc***)

	**Glycolysis/Gluconeogenesis (mmu00010)**	aldo-keto reductase family 1, member A1 (aldehyde reductase) (***Akr1a1***)
		aldolase A, fructose-bisphosphate (***Aldoa***)
		aldolase C, fructose-bisphosphate (***Aldoc***)
		enolase 1, alpha non-neuron (***Eno1***)
		glucose phosphate isomerase 1 (***Gpi1***)
		glyceraldehyde-3-phosphate dehydrogenase, pseudogene 15 (***Gapdh-ps15***)
		lactate dehydrogenase B (***Ldhb***)

	**Adherens junction (mmu04520)**	actin, beta (***Actb***)
		actin, gamma, cytoplasmic 1 (***Actg1***)
		catenin (cadherin associated protein), alpha 1 (***Ctnna1***)
		catenin (cadherin associated protein), beta 1 (***Ctnnb1***)
		cell division cycle 42 (***Cdc42***)
		protein tyrosine phosphatase, receptor type, B (***Ptprb***)
		sorbin and SH3 domain containing 1 (***Sorbs1***)

Downregulation	**Systemic lupus erythematous (mmu05322)**	H2A histone family, member Z (***H2afz***)
		cathepsin G (***Ctsg***)
		histone cluster 1, H2ac (***Hist1h2ac***)

*Gene abbreviations are indicated in bold.*

Taken together, results from microarray analysis showed that 1 μM luteolin treatment for 24 h induced differentiation of hNSCs and directed its fate toward astrocytes. Besides, these results also suggest that luteolin modulated the differentiation of hNSCs toward astrocytes *via* upregulation of astrogenic genes such as *GFAP, WNT*, and *JAK*, while it suppressed the neuronal and oligodendrocytes differentiation by downregulation of *NEUROG2*, *DNMT1*, *DNMT3B*, *NEUROD1*, *NEUROD6*, *NOTCH3*, *BMP7* and *TUBB3*, and *MBP*.

#### Luteolin Increased the Number of GFAP+ Cells After 24 h Incubation

**FIGURE 2:**
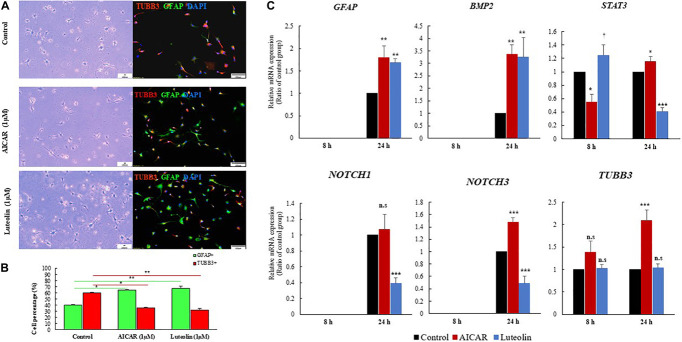
Luteolin treatment (1 μM) for 24 h promoted differentiation of hNSCs into astrocytes. **(A)**, Images of differentiated hNSCs treated with differentiation medium (Control), 1 μM of AICAR, or 1 μM of Luteolin fixed after 24 h and stained with TUBB3 (red), *GFHP* (green), and counterstained with DAPI (blue), unstained cells Scale bar = 100 nm, stained cells Scale bar = 100 μm, Magnification = ×10. **(B)** Quantification of GFAP and TUBB3 positive cells among total DAPI-positive cell. Data are expressed as mean ± SEM of two independent experiments. **(C)** RT-qPCR validation results obtained after 8 and 24 h of treatment with differentiation medium, 1 μM Luteolin or 1 μM AICAR. Data are expressed as mean ± S.D. of three independent experiments. ***p < 0.001, **p ≤ 0.01, *p < 0.05, and ^†^*p* < 0.1 (ANOVA followed by Dunnett’s *post hoc* test).

We performed immunostaining to quantify neuronal and glial cell populations after 24 h of differentiation induction in the presence or absence of luteolin and AICAR. It was previously reported that hNSCs fate is determined within 24 h of differentiation induction (Sasaki et al., 2019). The immunostaining of hNSCs showed that untreated control, and both AICAR- and luteolin-treated cells were exclusively differentiated into neurons and astrocytes. Only TUBB3^+^ and GFAP^+^ cells were detected after 24 h of incubation (Figure 2A). MBP^+^ cells were not detected within 24 h of differentiation induction with both control and treatment solutions (Data not shown). When treated with differentiation medium, 59.97% of hNSCs were differentiated into neurons (TUBB3^+^ cells) and 40.03% cells were differentiated into astrocytes (GFAP^+^ cells); however, 1 μM AICAR and 1 μM Luteolin treatments significantly decreased the percentages of TUBB3^+^ cells to respectively 36.66 and 33.2% (*p* < 0.05) and increased the percentages of astrocytes (GFAP^+^ cells) to 64.44 and 67.8%, respectively (*p* < 0.05). These data indicate that as AICAR treatment, luteolin treatment enhanced the differentiation of hNSCs into astrocytes (Figure 2B).

#### Luteolin Regulated Astrogliogenic Gene Expressions in Human Neural Stem Cells

We validated the expressions of astrogliogenic genes using RT-qPCR. As shown in Figure 2C, *GFAP* was not expressed in any treatment condition after 8 h even in repeated RT-qPCR experiments. However, after 24 h, *GFAP* was significantly overexpressed in AICAR-treated (relative *GFAP* mRNA expression = 1.8, *p* = *0.001*) and luteolin treated (relative *GFAP* mRNA expression = 1.69, *p* = *0.003*) cells compared to differentiation medium-treated control cells. This finding confirms that luteolin stimulated the differentiation of hNSCs into astrocytes.

Different signaling pathways, including BMP, JAK-STAT, Wnt, Notch, TGF-β, and FGF signaling, are known to regulate the expression *GFAP* gene, which, in turn, induces astrocytogenesis (Wen et al., 2009; Lee et al., 2015). Therefore, we evaluated the expressions of the *BMP2* as a representative gene of BMP signaling, and *NOTCH1* and *NOTCH3* as representative genes of Notch signaling. We also evaluated the expression of the transcription factor *STAT3*, which binds to the promoter of *GFAP* to induce its expression.

*BMP2* gene was not expressed after 8 h treatment in both controls and treated cells, while it was significantly overexpressed after 24 h treatment with 1 μM luteolin (Relative *BMP2* mRNA expression = 3.26, *p* = *0.00*3) and with the positive astrogenic inducer AICAR (Relative *BMP2* mRNA expression = 3.36, *p* = *0.00*2) (Figure 2C). *STAT3* was significantly overexpressed after 8 h in luteolin-treated hNSCs (Relative *STAT3* mRNA expression = 1.25, *p* = *0.059*) and it was significantly decreased after 24 h treatment (Relative *STAT3* mRNA expression = 0.41, *p* = *0.0001*) (Figure 2C). On the other hand, the AICAR treatment significantly decreased the STAT3 expression after 8 h treatment (Relative *STAT3* mRNA expression = 0.55, *p* = *0.005*), and it increased after 24 h treatment (Relative *STAT3* mRNA expression = 1.15, *p* = *0.03*) (Figure 2C).

*NOTCH1* and *NOTCH3* were not expressed in any treatment condition after 8 h (Figure 2C). When treated for 24 h with 1 μM luteolin, the expressions of *NOTCH1* and *NOTCH3* were significantly decreased to 0.39 and 0.48, respectively (*p* = *0.001* and *0.0001* respectively). When treated with 1 μM AICAR for the same duration (24 h), the expression of *NOTCH1* gene was unchanged and that of *NOTCH3* significantly increased to 1.48 (*p* = *0.001*). This result shows that unlike AICAR, luteolin inhibited Notch signaling and therefore inhibited the self-renewal of hNSC (Figure 2C).

#### Luteolin Treatment Did Not Affect Neurogenic Gene Expression in Human Neural Stem Cells

The validation of *TUBB3* gene expression using RT-qPCR showed that luteolin treatment for 8 and 24 h did not affect the expression of *TUBB3* in hNSCs (Relative *TUBB3* mRNA expression = 1.02, *p* = 0.92 and 1.03, *p* = *0.949* respectively for 8 and 24 h treatment) (Figure 2C). On the other hand, when treated with AICAR, the expressions of *TUBB3* in hNSCs were increased to 1.39 and 2.09, respectively, following 8 and 24 h of incubation (*p* = *0.21* and *p* = *0.0001* respectively) (Figure 2C) suggesting, thus, that AICAR is not a specific astrocytogenesis inducer.

#### Luteolin Treatment Inhibited Oligogenic Gene Myelin Basic Protein Expression in Human Neural Stem Cells

Our immunostaining results using the oligodendrocytes marker anti-MBP protein antibody showed that 1 μM luteolin treated hNSCs did not differentiate into oligodendrocytes within 24 h of differentiation induction (data not shown). In its turn, the global gene expression analysis shows that this treatment downregulates the MBP gene (FC = −1.37, *p* < *0.05*). The validation analysis of this gene expression using RT-qPCR also showed that hNSCs did not express the MBP gene after 24 h of luteolin treatment (Data not shown), confirming, therefore, that luteolin did not induce oligodendrocyte genesis of hNSCs.

### Results Obtained From the Animal Experiments

**FIGURE 3:**
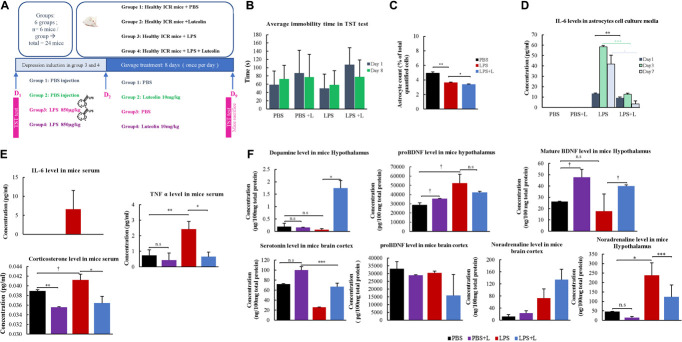
Behavioral and physiological effects of Luteolin treatment on LPS depression induced mice. **(A)** Experimental design. **(B)** Effect of the oral administration of luteolin (10 mg/kg) on LPS depression induced mice (LPS + L) immobility times in tail suspension test compared to the control (PBS), the EPS group untreated with luteolin (LPS) and to control group treated with luteolin (PBS + L) at day 1 and at day 8. Results are expressed in mean of immobility time ±SD. Paired *t*-test was applied to compare results of the same group between Day 1 and Day 8, no significant results were obtained, One-way ANOVA test was applied to compare between groups at Day 1 and at Day 8, no significant results were obtained. **(C)** Effect of luteolin treatment on astrocytes numbers. Results are expressed as mean of astrocytes percentage of three independent experiments ±SD. **(D)** Effect of luteolin treatment on IL-6 secretion by cultured primary at Day 1, Day 3, and Day 7. Results are expressed as mean concentration of IL-6 secreted in isolated primary astrocytes’ cell culture media of three independent experiments ±SD. **(E,F)** ELISA assays results. Data are expressed as mean of concentration ±S.D. of three independent experiments. *^∗∗∗^p* < 0.001, *^∗∗^p* ≤ 0.01, *^∗^p* < 0.05, and ^†^*p* < 0.1, n.s: not significant (ANOVA followed by Dunnett’s *post hoc* test).

Figure 3A shows the animal experiment design.

#### Effect of Luteolin Treatment on the Immobility Time in Lipopolysaccharide-Induced Depression Mice

Results from TST test indicate that the daily oral administration of luteolin (10 mg/kg body weight) slightly decreased the immobility time of LPS-induced depression mice on the 8th day of TST to 77.5 ± 52.5 s compared to the initial test performed on the 1st day with a value of 107.17 ± 41.1 s. It also decreased the immobility time of mice treated with PBS + L to 77.17 ± 35.5 s compared to the initial test at day 1 (86.83 ± 55.4 s). These decreases were statistically non-significant (Figure 3B).

#### Effect of Luteolin Treatment on Lipopolysaccharide Induced Depression Mice Astrocytes’ Number

In the present study, the number of isolated astrocytes from whole brain of the LPS-induced depression mice was compared to that of LPS-induced depression mice treated with luteolin and control mice. Results show that LPS significantly reduced the number of astrocytes (*p* = *0.008*), however, luteolin treatment didn’t restore the loss of astrocytes number (Figure 3C).

#### Effect of Luteolin Treatment on IL-6 Secretion by Lipopolysaccharide Induced Depression Mice Astrocytes’

The quantification of the pro-inflammatory cytokine IL-6 in the cell culture media of primary isolated astrocytes after 1, 3, and 7 days post *in vitro* isolation show that luteolin treatment didn’t affect the IL-6 level in PBS mice (PBS + L vs. PBS group), while LPS treatment significantly increase the production of IL-6 by astrocytes (LPS vs. PBS group), and luteolin treatment significantly decreased this pro-inflammatory cytokine secretion by astrocytes in LPS-induced depression mice (LPS + L vs. LPS group) (Figure 3D).

#### Luteolin Treatment Changed Depression’s Biomarkers Levels

We evaluated the level of serum and central nervous system depression’s biological markers in mice. Figure 3E shows that the oral administration of luteolin (10 mg/kg) changed the levels of the proinflammatory cytokines IL-6 and TNFα, and that of corticosterone in mice sera. IL-6 level was increased in LPS treated mice group compared to control group (PBS group), and luteolin treatment normalized this increase in LPS-induced depression mice (LPS + L group). Although statistically not significant (*p* > 0.05), the TNF-α level showed a decrease of 41.1% in control mice treated with luteolin (PBS + L) compared to untreated mice (PBS). LPS treatment significantly increased this pro-inflammatory cytokine level of 2.31% in mice sera compared to control mice (*p* = *0.01*), and a statistically significant decrease of this cytokine level (73.15%) was observed when LPS-induced depression mice were treated with luteolin (*p* = *0.04*) (Figure 3E). The corticosterone level showed a decrease of 8.49% in control mice treated with luteolin (PBS + L) compared to untreated mice (PBS) (*p* = *0.007*). LPS treatment significantly increased this stress hormone level of 0.06% compared to control mice (*p* = *0.1*), and luteolin treatment significantly decreased its level in LPS-induced depression mice (a decrease of 73.15% compared to untreated LPS-induced depression mice, *p* = *0.05*).

The oral administration of luteolin (10 mg/kg) also significantly changed the levels of the mature BDNF, the noradrenaline and the dopamine in the hypothalamus of LPS-induced depression mice, and it didn’t significantly affect the level of pro-BDNF. Likewise, it didn’t change (*p* > 0.05) the levels of pro-BDNF, serotonin and noradrenaline in the cerebral cortex of LPS-induced depression mice model (Figure 3F).

#### Luteolin Treatment Affected Global Gene Expression in Neural Stem Cells of Lipopolysaccharide-Induced Depression Model

**FIGURE 4:**
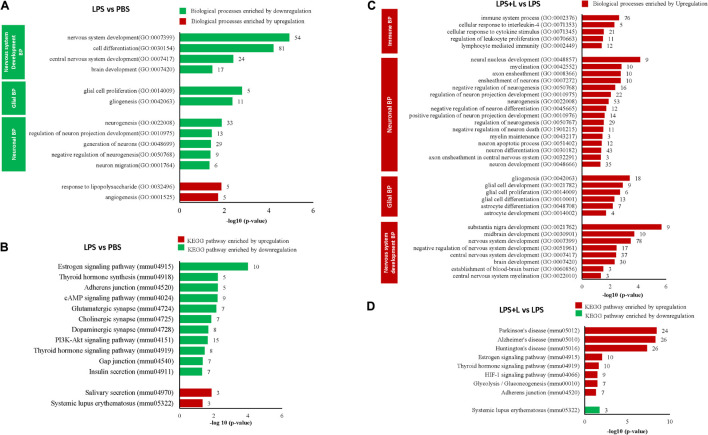
Luteolin treatment significantly regulated gene expression in NSCs of LPS-induced depression mice model. **(A,B)** Differentially regulated biological processes (BP) and top significantly enriched KEGG signaling pathways in NSCs of LPS-induced depression mice (LPS) compared to untreated mice (PBS). **(C,D)** Differentially regulated biological processes (BP) and top significantly enriched KEGG signaling pathways in NSCs of LPS-induced depression mice treated with luteolin (LPS + L) compared to untreated LPS-induced depression mice (LPS).

We evaluated the effect of LPS treatment on global genes expression in the NSCs of the chosen depression animal model (LPS-induced depression mice) by evaluating the transcriptomic changes in NSCs. Results show that LPS injection (850 μg/kg) significantly regulated 382 genes (−1.3 < Fold change < 1.3; *p* < 0.05). It downregulated 325 genes and upregulated 57 genes compared to untreated mice (PBS group) (data not shown). Five neuronal biological processes, namely neuron migration (GO:001764), negative regulation of neurogenesis (GO:0050768), generation of neurons (GO:0048699), regulation of neuron projection development (GO:0010975), and neurogenesis (GO:0022008); two glial biological processes particularly the gliogenesis (GO:0042063) and glial cell proliferation (GO:0014009); and four development biological processes related to brain development (GO:0007420), central nervous system development (GO:0007417), cell differentiation (GO:0030154) and nervous system development (GO:0007399), were enriched by downregulation (Figure 4A). The 33 downregulated genes related to neurogenesis are *Ddx6*, *Rab3a*, *Actb*, *Apcdd*, *Apoe*, *B2m*, *Camk2a*, *Ctnnb1*, *Clu*, *Dbi*, *Dynlt1f*, *Eef1a1*, *Fn1*, *Gja1*, *Gstp1*, *Gpm6a*, *Hspb1, Hsp90aa1*, *H2-D1*, *H2-K1, Id3*, *Itm2c*, *Ppia*, *Pcnt*, *Plpp3*, *Ptn*, *Prom1*, *Rpl4*, *Serpine2, Slc1a3, Sod1, Ywhae*, and *Vim*, and the 11 genes related to gliogenesis include the *Apcdd1, Ctnnb1*, *Clu*, *Dbi*, *Fn1*, *Gstp1*, *Plpp3*, *Ptn*, *Serpine2*, *Sod1*, and *Vim*. Besides, two biological processes related to angiogenesis (GO:0001525) and to response to polysaccharide (GO:0032496) were enriched by upregulation (Figure 4A). The LPS injection significantly regulated genes related to different KEGG signaling pathways (Figure 4B). The estrogen signaling pathway (mmu04915), with ten genes being regulated, was the most significantly KEGG signaling enriched by downregulation (*p* = 9.87E-05). The downregulated genes include the *Fos*, *Gnas*, *Creb3*, *Gnai2*, *Hspa1b*, the *Hspa8*, *Hsp90ab1*, *Hsp90aa1*, *Hsp90b1* and the *Pik3cd* (Figure 4B and Table 1). The KEGG signaling pathways, Thyroid hormone synthesis (mmu04918), Adherens junction (mmu04520), cAMP signaling pathway (mmu04024), glutamatergic and dopaminergic synapses (mmu04724, mmu04728), PI3K-AKT signaling pathway (mmu04151), the thyroid hormone signaling pathway (mmu04919), gap junction (mmu04540) and insulin secretion (mmu04911) were also enriched by downregulation (Figure 4B). Details about regulated genes are mentioned in Table 1.

We also evaluated the effect of oral administration of luteolin (10 mg/kg body weight) on the transcriptomic changes in NSCs of LPS-induced depression mice. The obtained results show that treatment with luteolin could significantly regulate the expression of 687 genes in NSCs of this animal model (−1.3 < Fold change < 1.3; *p* < 0.05) compared to untreated depression model mice (LPS + L group vs. LPS group), with upregulation of 642 genes and downregulation of 45 genes (data not shown). Top 10 significantly up and downregulated genes and their functions are presented in Supplementary Tables 6, 7, respectively. The upregulated genes significantly enriched biological processes related to nervous system development, glial and neuronal processes as well as signaling biological processes (Figure 4C). Interestingly, in these induced depression mice neural stem cells’, the luteolin treatment upregulated 53 and 18 genes related to the neurogenesis and gliogenesis biological processes respectively. The genes related to neurogenesis biological process are: the *Arf1, Ddx6*, *Ndrg1*, *Rab3a*, *Actb*, *Apcdd1, App*, *Apoe*, *B2m*, *Ctnna1*, *Ctnnb1*, *Cav1*, *Cdc42*, *Clu*, *Dbi*, *Dynlt1f*, *Eef1a1*, *Eef2, Eif4g2*, *Fn1*, *Gja1, Gstp1*, *Gpm6a*, *Hey1*, *Hspb1*, *Hsp90aa1*, *Hexa*, *H2-D1*, *H2-K1*, *Id3, Id4*, *Itm2c*, *Il2*, *Mif*, *Mt2*, *Mt*), *Mgll*, *Ntrk2*, *Ppia*, *Pcnt*, *Pmp22*, *Plpp3*, *Ptn*, *Prom1*, *Plp1*, *Rpl4*, *Serpine2*, *Slc1a3*, *Slc9a3r1*, *Snx3*, *Sod1*, *Ywhae*, and *Vim*, while those modulating the gliogenesis biological process are: *Ndrg1*, *Apcdd1*, *Ctnnb1*, *Clu*, *Dbi*, *Eef2*, *Fn1*, *Gstp1*, *Id4*, *Mt2*, *Mt3*, *Ntrk2*, *Plpp3*, *Ptn*, *Plp1*, *Serpine2*, *Sod1*, *Vim*. The regulated genes also significantly modulated different KEGG signaling pathways (Figure 4D). The KEGG signaling pathway Parkinson’s disease (mmu05012) was the most significantly enriched signaling pathway by upregulation (*p* = 3.53E-09). Twenty-four genes of this signaling pathway were enriched by upregulation. It includes 5 genes modulating the ATP synthase, H + transporting mitochondrial complexes (*Atp5g3*, *Atp5h*, *Atp5o*, *Atp5e*, *Atp5c1*), 8 genes of NADH dehydrogenase (*Ndufa13*, *Ndufa3, Ndufa9, Ndufb11, Ndufb5, Ndufv3, LOC102641347, ND5)*, 6 genes of cytochrome c oxidase subunits (*Cox4i1, Cox7a2, Cox7a2l, Cox6a1, Cox5a, Cox5b, Cox6b), as well as* guanine *Gnai2*, *Slc25a4*, *Slc25a5*, and *Uqcrb*. Also, twenty-six genes in Alzheimer’s disease KEGG signaling pathway (mmu05010, *p* = 4.76E-09), 26 genes in Huntington’s disease KEGG signaling pathway (mmu05016, *p* = 4.77E-08), 17 genes in Non-alcoholic fatty liver disease (NAFLD) KEGG signaling pathway (mmu04932, *p* = 0.0001), 10 in Estrogen signaling pathway (mmu04915, *p* = 0.0093) KEGG signaling pathway, 10 genes in thyroid hormone signaling pathway (mmu04919; *p* = 0.02), 9 genes in HIF-1 signaling pathway (mmu04066, *p* = 0.03), 7 genes glycolysis/gluconeogenesis (mmu00010, *p* = 0.03), and 7 genes in adherens junction (mmu04520, *p* = 0.04) were also enriched by upregulation. Systemic lupus erythermatosus (mmu05322, *p* = 0.017), was the only KEGG signaling pathway enriched by downregulation. Details about regulated genes are mentioned in Table 2.

Ultimately, we evaluated the effect of oral luteolin treatment (10 mg/kg body weight) on the transcriptomic changes in NSCs of normal mice (PBS + L group vs. PBS group, Supplementary Figure 4). The obtained results point out the modulation of gene expression by luteolin treatment in NSCs of normal mice (−1.3 < Fold change < 1.3; *p* < 0.05) compared to untreated normal mice, with upregulation of 314 genes and downregulation of 428 genes (data not shown). Moreover, we found that luteolin treatment significantly downregulated 4 glial biological processes, namely the glial cell migration (GO: 008347), negative regulation of gliogenesis (GO:0014014), glial cell differentiation (GO:0010001), and negative regulation of oligodendrocyte differentiation (GO:0048715) in NSCs of normal mice (PBS + L group), while it downregulated 8 neuronal biological processes, up regulated 9 others (Supplementary Figure 4A). In this mice group NSCs’, the effect of luteolin treatment on glial biological processes was more pronounced than that of the neurogenic effect and it was revealed by the downregulation of three genes related to the negative regulation of oligodendrocytes differentiation (*Ctnnb1*, *Notch1*, and *Sirt2*), the downregulation of 9 genes involved in the glial cell differentiation (*Ndrg1*, *Ctnnb1*, *Clu*, *Notch1*, *Plpp3*, *Serpine2*, *Sirt2*, *Sod1*, *Vim*), the downregulation of 4 genes related to the negative regulation of gliogenesis (*Ctnnb1*, *Notch1*, *Ptn*, *Sirt2*) and the downregulation of the 4 genes involved in the glial cell migration (*Apcdd1, Ctnnb1*, *Fn1*, *Vim*). The luteolin treatment also enriched 8 KEGG signaling pathway by downregulation and only one KEGG signaling by upregulation (Supplementary Figure 4B). The enriched KEGG signaling by upregulation was the Wnt KEEG signaling pathways (mmu04310, *p* = 0.04) with 6 genes being upregulated (*Apc2*, *Camk2a*, *Ccnd2*, *Prickle2*, *Tcf7l1*, *Wnt11*). The KEGG signaling pathways enriched by downregulation were Hippo signaling pathway (mmu04390, *p* = 3.24E-02, 9 genes), tight junction (mmu04530, *p* = 2.31E-02, 7 genes), thyroid hormone signaling pathway (mmu04919, *p* = 0.022, 8 genes), gap junction (mmu04540, *p* = 0.019, 7 genes), HIF-1 signaling (mmu04066, *p* = 0.012, 8 genes), dopaminergic synapse (mmu04728, *p* = 0.005, 10 genes), Alzheimer’s disease (mmu05010, *p* = 0.0004, 14 genes), and Parkinson’s disease (mmu05012, *p* = 0.0003, 13 genes) (Supplementary Figure 4B). Details about regulated genes are mentioned in Supplementary Table 8.

#### Luteolin Treatment Affected Global Gene Expression in Hippocampus of Lipopolysaccharide-Induced Depression Model

**FIGURE 5:**
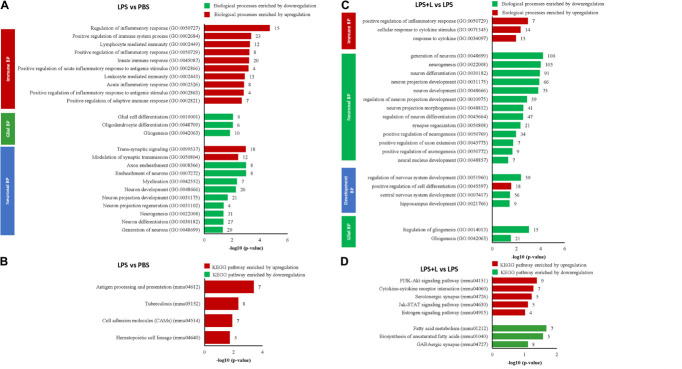
Luteolin treatment significantly regulated gene expression in hippocampus of LPS-induced depression mice model. **(A,B)** Differentially regulated biological processes (BP) and top significantly enriched KEGG signaling pathways in hippocampus of LPS-induced depression mice (LPS) compared to untreated mice (PBS). **(C,D)** Differentially regulated biological processes (BP) and top significantly enriched KEGG signaling pathways in hippocampus of LPS-induced depression mice treated with luteolin (LPS + L) compared to untreated LPS-induced depression mice (LPS).

We used DNA microarray to study the transcriptomic changes in the hippocampus of the depression mice model. Results show that LPS injection (850 μg/kg) significantly regulated 665 genes (−1.3 < Fold change < 1.3; *p* < 0.05). It downregulated 348 genes and upregulated 317 genes in this animal model of depression (LPS group mice) compared to untreated mice (PBS group) (data not shown). Nine neuronal biological processes including neuron differentiation (GO:0030182), neurogenesis (GO:0022008) were significantly downregulated. Ten genes related to gliogenesis (GO:0042063), 6 genes related to oligodendrocyte differentiation (GO:0048709), and 9 genes related to glial cell differentiation (GO:0010001) were also significantly downregulated. However, ten immune biological processes were significantly upregulated (Figure 5A). Thirty-one genes related to the neurogenesis biological process were downregulated. It includes the *Atp7a*, *Bcl11a*, *C1qtnf5*, *Cd9*, *Ndrg1*, *Rufy3*, *Abi2*, *Aspa*, *Camk2a*, *Ctnna1*, *Col25a1*, *Cdkn1c*, *Ddr1*, *Enpp2*, *Folr1*, *Hspa5*, *Lpar1*, *Micall1*, *Mapk8*, *Miat*, *Myo7a*, *Ntn4*, *Otx2*, *Prex2*, *Plp1*, *Rtn4*, *Robo3*, *Six3*, *Slc11a2*, *Stx3* and the *Tspo*. Equally, in these mice hippocampus, the LPS injection downregulated 10 genes related to gliogenesis (*Cd9, Ndrg1*, *Aspa*, *Enpp2*, *Lpar1*, *Miat*, *Otx2*, *Plp1*, *Rtn4* and *Tspo*). LPS injection also significantly enriched KEEG signaling pathways by upregulation (Figure 5B). It includes the hematopoietic cell lineage (mmu04640, *p* = 0.018, 5 genes), cell adhesion molecules (CAMs) (mmu04514, *p* = 0.012, 7 genes), tuberculosis (mmu05152, *p* = 0.004,8 genes), and antigen processing and presentation (mmu04612, *p* = 3.95E-04, 7 genes). Details about regulated genes are presented in Table 3.

**TABLE 3:** Top significantly enriched KEGG signaling pathways in Hippocampus of LPS-induced depression mice (LPS) compared to untreated normal mice (PBS).

Regulation	KEGG signaling	Genes
Upregulation	**Hematopoietic cell lineage (mmu04640)**	CD44 antigen (***Cd44***)
		Fc receptor, IgG, high affinity I (***Fcgr1***)
		colony stimulating factor 1 receptor (***Csf1r***)
		histocompatibility 2, class II antigen E beta (***H2-Eb1***)
		sialic acid binding Ig-like lectin H (***Siglech***)

	**Cell adhesion molecules (CAMs) (mmu04514)**	H-2 class I histocompatibility antigen, K-D alpha chain (***LOC101056305***)
		cadherin 15 (***Cdh15***)
		contactin 2 (***Cntn2***)
		golgi apparatus protein 1 (***Glg1***)
		histocompatibility 2, K1, K region (***H2-K1***)
		histocompatibility 2, Q region locus 6 (***H2-Q6***)
		histocompatibility 2, class II antigen E beta (***H2-Eb1***)

	**Tuberculosis (mmu05152)**	CD74 antigen (invariant polypeptide of major histocompatibility complex, class II antigen-associated) (***Cd74***)
		Fc receptor, IgE, high affinity I, gamma polypeptide (***Fcer1g***)
		Fc receptor, IgG, high affinity I (***Fcgr1***)
		Fc receptor, IgG, low affinity III (***Fcgr3***)
		apoptotic peptidase activating factor 1 (***Apaf1***)
		calcium/calmodulin-dependent protein kinase II, delta (***Camk2d***)
		cathepsin S (***Ctss***)
		histocompatibility 2, class II antigen E beta (***H2-Eb1***)

	**Antigen processing and presentation (mmu04612)**	CD74 antigen (invariant polypeptide of major histocompatibility complex, class II antigen-associated) (***Cd74***)
		H-2 class I histocompatibility antigen, K-D alpha chain (***LOC101056305***)
		beta-2 microglobulin (***B2m***)
		cathepsin S (***Ctss***)
		histocompatibility 2, K1, K region (***H2-K1***)
		histocompatibility 2, Q region locus 6 (***H2-Q6***)
		histocompatibility 2, class II antigen E beta (***H2-Eb1***)

*Gene abbreviations are indicated in bold.*

We further used DNA microarray to study the transcriptomic changes in the hippocampus of the depression mice model after oral administration of luteolin (10 mg/kg body weight). Results show that treatment with luteolin significantly regulated the expression of 1371 genes in hippocampus of this animal model (−1.3 < Fold change < 1.3; *p* < 0.05) compared to untreated depression model mice (LPS + L group vs. LPS group), with upregulation of 324 genes and downregulation of 1047 genes (Data not shown). Top 10 significantly up and downregulated genes and their functions are presented in Supplementary Tables 9, 10, respectively. The upregulated genes significantly enriched biological processes related to different immune responses namely; response to cytokine (GO:0034097), cellular response to cytokine stimulus (GO:0071345) and positive regulation of inflammatory response (GO:0050729) as well as to the development biological process positive regulation of cell differentiation (GO:0045597) (Figure 5C). The downregulated genes significantly enriched 13 neuronal biological processes; mainly generation of neurons (GO:0048699, 100 genes), neurogenesis (GO:0022008, 105 genes), neuron differentiation (GO:0030182, 91 genes), 3 development processes including regulation of nervous system development (GO:0051960, 59 genes), central nervous system development (GO:0007417, 56 genes) and hippocampus development (GO:0021766), and 2 glial processes namely regulation of gliogenesis (GO:0014013, 15 genes) and gliogenesis (GO:0042063, 21 genes) (Figure 5C). The regulated genes also significantly enriched different KEEG signaling pathways (Figure 5D and Table 4). Luteolin treatment upregulated 9 genes involved in PI3K-Akt signaling (mmu04151, *p* = 0.04) namely the *Col2a1*, *Egfr, Fgf18*, *Il4ra*, *Lama5*, *Pik3cg*, *Prlr*, *Rxra*, and the *Rps6*; 7 genes in cytokine-cytokine receptor interaction (mmu04060, *p* = 0.05) including the *Relt*, *Ccr2*, *Eda*, *Inhbb*, *Il17ra, Il4ra*, and the *Prlr*; 5 genes in serotonergic synapse (mmu04726, *p* = 0.05) namely the *Htr5b*, *Gnas*, *Alox12*, *Alox15, Alox8*; 5 genes in Jak-STAT signaling (mmu04630, *p* = 0.07) including the *Crebbp Il4ra*, *Pik3cg, Prlr*, and the *Stat5b*; and 4 genes in Estrogen signaling pathway (mmu04915, *p* = 0.096) including the *Elovl5*, *Acaa1a*, *Acaa2, Acsl3*, *Acadl*, *Elovl2*, and the *Fads2*. Besides, luteolin treatment downregulated 7 genes in fatty acid metabolism KEEG signaling (mmu012, *p* = 0.02) particularly the *Elovl5*, *Acaa1a*, *Acaa2*, *Acsl3*, *Acadl*, *Elovl2*, *Fads2*; 5 genes in biosynthesis of unsaturated fatty acids (mmu01040, *p* = 0.02) including the *Elovl5*, *Acaa1a, Acot1*, *Elovl2*, *Fads2*, and 8 genes in GABAergic synapse (mmu04727, *p* = 0.07) namely the *Gabrb3*, *Gphn*, *Gad1, Glul*, *Gnai3*, *Gnb1*, *Gng2*, and the *Prkacb* (Figure 5D and Table 4).

**TABLE 4:** Top significantly enriched KEGG signaling pathways in Hippocampus of LPS-induced depression mice treated with luteolin (LPS + L) compared to untreated LPS-induced depression mice (LPS).

Regulation	KEGG signaling	Genes
Upregulation	**PI3K-Akt signaling pathway (mmu04151)**	collagen, type II, alpha 1 (***Col2a1***)
		epidermal growth factor receptor (***Egfr***)
		fibroblast growth factor 18 (***Fgf18***)
		interleukin 4 receptor, alpha (***Il4ra***)
		laminin, alpha 5 (***Lama5***)
		phosphoinositide-3-kinase, catalytic, gamma polypeptide (***Pik3cg***)
		prolactin receptor (***Prlr***)
		retinoid X receptor alpha (***Rxra***)
		ribosomal protein S6 (***Rps6***)

	**Cytokine-cytokine receptor interaction (mmu04060)**	RELT tumor necrosis factor receptor (***Relt***)
		chemokine (C-C motif) receptor 2 (***Ccr2***)
		ectodysplasin-A (***Eda***)
		inhibin beta-B (***Inhbb***)
		Interleukin 17 receptor A (***Il17ra***)
		Interleukin 4 receptor, alpha (***Il4ra***)
		prolactin receptor (***Prlr***)

	**Serotonergic synapse (mmu04726)**	5-hydroxytryptamine (serotonin) receptor 5B (***Htr5b***)
		GNAS (guanine nucleotide binding protein, alpha stimulating) complex locus (***Gnas***)
		arachidonate 12-lipoxygenase (***Alox12***)
		arachidonate 15-lipoxygenase (***Alox15***)
		arachidonate 8-lipoxygenase (***Alox8***)

	**Jak-STAT signaling pathway (mmu04630)**	CREB binding protein ***(Crebbp***)
		interleukin 4 receptor, alpha (***Il4ra***)
		phosphoinositide-3-kinase, catalytic, gamma polypeptide (***Pik3cg***)
		prolactin receptor (***Prlr***)
		signal transducer and activator of transcription 5B (***Stat5b***)

	**Estrogen signaling pathway (mmu04915)**	FK506 binding protein 4 (***Fkbp4***)
		GNAS (guanine nucleotide binding protein, alpha stimulating) complex locus (***Gnas***)
		epidermal growth factor receptor (***Egfr***)
		phosphoinositide-3-kinase, catalytic, gamma polypeptide (***Pik3cg***)

Downregulation	**Fatty acid metabolism (mmu01212)**	ELOVL family member 5, elongation of long chain fatty acids (yeast) (***Elovl5***)
		acetyl-Coenzyme A acyltransferase 1A (***Acaa1a***)
		acetyl-Coenzyme A acyltransferase 2 (mitochondrial 3-oxoacyl-Coenzyme A thiolase) (***Acaa2***)
		acyl-CoA synthetase long-chain family member 3 (***Acsl3***)
		acyl-Coenzyme A dehydrogenase, long-chain (***Acadl***)
		elongation of very long chain fatty acids (FEN1/Elo2, SUR4/Elo3, yeast)-like 2 (***Elovl2***)
		fatty acid desaturase 2 (***Fads2***)

	**Biosynthesis of unsaturated fatty acids (mmu01040)**	ELOVL family member 5, elongation of long chain fatty acids (yeast) (***Elovl5***)
		acetyl-Coenzyme A acyltransferase 1A (***Acaa1a***)
		acyl-CoA thioesterase 1 (***Acot1***)
		elongation of very long chain fatty acids (FEN1/Elo2, SUR4/Elo3, yeast)-like 2 (***Elovl2***)
		fatty acid desaturase 2 (***Fads2***)

	**GABAergic synapse (mmu04727)**	gamma-aminobutyric acid (GABA) A receptor, subunit beta 3 (***Gabrb3***)
		gephyrin (***Gphn***)
		glutamate decarboxylase 1 (***Gad1***)
		glutamate-ammonia ligase (glutamine synthetase) (***Glul***)
		guanine nucleotide binding protein (G protein), alpha inhibiting 3 (***Gnai3***)
		guanine nucleotide binding protein (G protein), beta 1 (***Gnb1***)
		guanine nucleotide binding protein (G protein), gamma 2 (***Gng2***)
		protein kinase, cAMP dependent, catalytic, beta (***Prkacb***)

*Gene abbreviations are indicated in bold.*

Ultimately, we evaluated the effect of oral luteolin treatment (10 mg/kg body weight) on the transcriptomic changes in hippocampus of normal mice (PBS + L group vs. PBS group) using DNA microarray (Supplementary Figures 4C,D). The obtained results point out the modulation of gene expression by luteolin treatment in hippocampus of normal mice (−1.3 < Fold change < 1.3; *p* < 0.05) compared to untreated normal mice, with upregulation of 381 genes and downregulation of 978 genes (Data not shown). Luteolin treatment significantly downregulated 5 immune biological processes, including inflammatory response (GO:0006954, 18 genes), 6 glial processes including gliogenesis (GO:0042063, 13 genes), glial cell differentiation (GO:0010001), oligodendrocyte differentiation (GO:0048709), and 4 neuronal processes namely neuron projection development (GO:0031175, 21 genes), neuron differentiation (GO:0030182, 29 genes), neurogenesis (GO:0022008, 38 genes) and myelination (GO:0042552, 8 genes) (Supplementary Figure 4C). Besides, luteolin treatment significantly upregulated the expression of 18 genes related to gliogenesis (GO:0042063) including the *Phgdh, Kras*, *Adgrg1*, *Atxn1*, *Ctnnb1*, *Dcx*, *Egr2*, *Fgfr3*, *Gfap*, *Mecp2*, *Nf2*, *Ntrk2*, *Ntrk3*, *Pten*, *Pdgfb*, *Synj1*, *Tenm4*, *Vtn*, while it downregulated 13 others (*Ndrg1, Apcdd1*, *Ctnnb1*, *Clu*, *Dbi*, *Fn1*, *Notch1*, *Plpp3*, *Ptn*, *Serpine2*, *Sirt2*, *Sod1*, and *Vim*), as well as different neuronal processes, particularly, neurogenesis (GO:0022008, 118 genes), neuron differentiation (GO:0030182, 101 genes), positive regulation of neurogenesis (GO:0050769, 40 genes), and positive regulation of neuron differentiation (GO:0045666) (Supplementary Figure 4C).

Along with other KEEG signaling pathways presented in Supplementary Figure 4D, dopaminergic synapse (mmu04728, *p* = *0.08*, 9 genes), GABAergique synapse (mmu04727, *p* = *0.07*, 7 genes), neurotrophin signaling pathway (mmu04722, *p* = 0.05, 9 genes), hippo signaling pathway (mmu04390, *p* = *0.013*, 12 genes), PI3K Akt signaling pathway (mmu04151, *p* = *0.008*, 22 genes) and signaling pathways regulating pluripotency of stem cells (mmu04550, *p* = 0.007, 12 genes), were significantly upregulated (Supplementary Figure 4D). Details about regulated genes are mentioned in Supplementary Table 11.

## Discussion

The present study is the first to report the effects of the natural flavonoid luteolin on hNSCs fate determination highlighting; therefore, its potential beneficial use, especially as an astrogliogenesis promoting compound. We have evaluated the effect of luteolin on the fate choice of hNSCs isolated from the fetal cortex. In terms of the therapeutic potential of NSCs in neurodegenerative diseases and neural injury, both fetal and adult brain-derived hNSCs have shown promising effects (Casarosa et al., 2014). However, fetal brain-derived NSCs grown as neurospheres can better mimic the brain developmental processes, including trilineage differentiation, proliferation, apoptosis, and migration (Schmuck et al., 2017). We found that luteolin could regulate the expression of the astrogenic gene *GFAP*, along with genes involved in WNT-β-catenin-BMP2-STAT3 pathways, which have been implicated in astrocytogenesis. In addition, an increased number of GFAP+ cells observed in immunostaining confirms the astrogenesis-inducing effects of luteolin on hNSCs. Besides, we compared our findings with a well-known positive astrogenic inducer AICAR, which further signifies the astrocyte-specific differentiation-inducing effects of luteolin.

The present study is also the first study to evaluate whether luteolin may exert an antidepressant effect by directing the fate choice of mice NSCs into astroglial and neuronal cells, restoring, therefore, the brain cells loss in the neuroinflammatory model of depression (LPS-induced depression mice). Numerous studies reported that a modification of astrocytes in the frontolimbic regions like the hippocampus, amygdala and ventral striatum is associated with depression. Likewise, most major depressed patients’ post-mortem brain analyses reported a decreased number of astrocytes in frontolimbic structures (Altshuler et al., 2010; Rajkowska and Stockmeier, 2013; Peng et al., 2015; Rial et al., 2016). The causality between astrocytic dysfunction and depression was also provided by animal studies showing that the selective destruction of frontocortical astrocytes is sufficient to induce depressive behavior (Banasr and Duman, 2008). Moreover, several studies evidence the reduced neurogenesis in animal models of depression and postmortem studies of depressed patients (Huffman and Taylor, 2020). Our results point out that luteolin treatment may exert an antidepressant effect in LPS mice by decreasing IL-6 production by astrocytes, decreasing the levels of IL-6, TNF alpha, and corticosterone in serum, and by increasing mature BDNF, dopamine and noradrenaline levels in the hypothalamus. Moreover, we found that luteolin treatment significantly regulated global genes expression, biological processes, and KEEG signaling in the isolated NSC and the hippocampi of the LPS induced depression mice model. It regulated gliogenesis and neurogenesis processes in the isolated mice’ NSCs and the hippocampi with a clearer gliogenesis upregulation observed in NSCs, highlighting then the potential use of luteolin as a neuro-glial enhancer to overcome depression.

Gene ontology enrichment analysis of differentially expressed genes between luteolin-treated and untreated control hNSCs showed that the most significantly enriched biological process was cell differentiation, followed by, but not limited to, neurogenesis, neuron development, neuron projection development, brain development, and regulation of astrocyte differentiation. When we averaged the signal intensities of genes related to astrogenesis-specific biological processes, we found that astrocyte differentiation- and positive regulation of astrocyte differentiation-related genes had higher signal intensity in luteolin-treated hNSCs compared to control cells, whereas negative regulation of astrocyte differentiation-related genes had lower signal intensity. Several KEGG signaling pathways, namely NOTCH, FOXO, TNF, p53, Hippo, dopaminergic synapse, and neurotrophin pathways, were also significantly regulated.

Considering previously published studies on genes involved in neural stem cell differentiation, we evaluated the effect of luteolin on 37 selected genes. We found similar expression patterns of these genes in luteolin-treated hNSCs (Hsieh et al., 2004; Cahoy et al., 2008; Yao and Jin, 2014; Hsieh and Zhao, 2016; Bejoy et al., 2019). Our results were marked by the downregulation of the stemness genes *NOTCH1* and *NOTCH3* (Venkatesh et al., 2017), neurogenic genes *DNMT1*, *DNMT3B*, *NEUROG2*, *BCL11B*, *NEUROD1*, *NEUROD1, NEUROD6, NOTCH1*, *NOTCH3*, and *TUBB3* (Yao and Jin, 2014), and oligodendrocyte genesis gene *MBP*. Besides, our findings were also marked by the upregulation of the astrogenic gene *GFAP* as well as other genes of the key signaling pathways involved in astrogenesis such as Wnt, BMP, and JAK-STAT pathways (Takouda et al., 2017). It is worth noting that DNA methylation, one of the core epigenetic modifications, has been implicated in several extrinsic pathways during neurogenesis both in physiologic and in diseased conditions (Wang et al., 2016b). *DNMT1* and *MECP2* are the key regulators of DNA methylation that control the timing and magnitude of astroglial differentiation. Conditional deletion of *DNMT1* in neural progenitor cells increases the expressions of astrocyte marker genes and activates the gliogenic JAK-STAT pathway (Fan et al., 2005). *MECP2* binds to the highly methylated regions of astrocyte-specific genes, such as *GFAP*, and suppresses their expression (Fan et al., 2005), inhibits astrocyte differentiation, and promotes neuronal differentiation (Kohyama et al., 2008; Tsujimura et al., 2009); whereas, loss of *MECP2* elevates the expression of glial markers and accelerates astrogenesis (Okabe et al., 2012; Forbes-Lorman et al., 2014). On the other hand, *TET*, an enzyme responsible for DNA demethylation, has been shown to negatively regulate neuronal differentiation in neuroblastoma cell line independent of its enzymatic activity (Gao et al., 2016). TETs also downregulate the expressions of *DNMT1* and *de novo* methyltransferases DNMT3A and DNMT3B and increase expression of the neurotrophic factor BDNF (Santiago et al., 2014). In this present study, we found that 24 h treatment with luteolin in hNSCs suppressed the expressions of *DNMT1*, *DNMT3B*, *MECP2*, while increased the expressions of Tet methylcytosine dioxygenase 1 (*TET1*), *JAK2*, *STAT4*, *STAT6*, and Brain-derived neurotrophic factor (*BDNF*). Besides, we also found that luteolin treatment could upregulate the expression of *NEUROG1*, whereas downregulate the expression of *NEUROG2*. *NEUROG*s are the proneural genes, which encode basic-helix-loop-helix (bHLH) transcription factors. Although both *NEUROG1* and *NEUROG2* individually promote neurogenesis, *NEUROG1* exerts a non-canonical role through inhibiting the proneural activity of *NEUROG2*, thus, in turn, induces the expression of negative regulators of neurogenesis, and represses the expression of positive regulators of neurogenesis, such as *NEUROD*s (Han et al., 2018). Ectopic expression of *NEUROD1*, another important bHLH transcription factor that mediates neuronal fate specification, has been reported to be sufficient to initiate a neurogenic program that closely recapitulates neuronal development *in vivo* (Pataskar et al., 2016). In our study, luteolin treatment repressed the expression of *NEUROD1* as well as *NEUROD 6*. In addition, luteolin treatment downregulated the expression of B-cell CLL/lymphoma 11 B (*BCL11b*), an important zinc finger protein transcription factor implicated in neurogenesis and in a pathological pathway of negative regulation of *BDNF* (Tang et al., 2011; Lennon et al., 2017). Thus, altogether our DNA microarray results suggest that luteolin treatment could provide an efficient platform for astrocyte differentiation from hNSCs, while negatively regulating the hNSCs self-renewal, neurodifferentiation, and oligodendrocytes genesis.

To validate the astrogenic effect of luteolin on hNSCs, immunocytochemistry was performed on 24 h luteolin-treated hNSCs. Results were compared to that of the positive astrogenic inducer AICAR-treated hNSCs and the untreated control hNSCs. After 24 h of differentiation induction, hNSCs were exclusively differentiated toward Tubb3+ and GFAP+ cells corresponding to neurons and astrocytes, respectively. We found that GFAP+ cells were increased in luteolin-treated hNSCs compared to untreated control hNSCs. This increase in the number of GFAP+ cells was even slightly higher in luteolin-treated cells than that observed in AICAR-treated cells. Conversely, the number of TUBB3+ cells was slightly lower in luteolin-treated cells compared to AICAR-treated cells. This finding suggests that luteolin would have a more powerful astrogenic effect on hNSCs than AICAR. Besides, MBP^+^ cells were not detected within 24 h of differentiation induction with both control and treatment solutions. As Grinspan et al., reported that glial differentiation takes place after neuronal differentiation and that astrocytes arise earlier than oligodendrocytes, our findings suggest that 24 h treatment was not enough to evaluate the effect of luteolin and AICAR on the oligodendrocytes differentiation process (Grinspan, 2002).

Previous studies reported that six signaling pathways, namely Wnt, BMP, Stat3, Notch, Shh, PDGF/EGF, ERK1/2, and JAK/STAT3 are involved in stem cell fate-determining signaling (Wen et al., 2009; Lee et al., 2015; Sasaki et al., 2019). To validate the findings of DNA microarray analysis and immunostaining, we further opted for quantitative genetic analysis using the RT-qPCR method. The expression profiles of seven genes, namely the neuron*-*specific *TUBB3*, the astrocyte-specific *GFAP*, the oligodendrocyte-specific *MBP* genes as well as the *BMP2*, *STAT3*, *NOTCH1*, and *NOTCH3* were analyzed to elucidate the molecular mechanism followed by luteolin to suppress the self-renewal properties of hNSCs, to attenuate the neurogenesis and to enhance the astrocytic differentiation in hNSCs. Additionally, the effects of AICAR on the expression of the aforementioned genes were also evaluated to compare the molecular mechanism followed by both molecules. The RT-qPCR results of 24 h post-treatment showed that both luteolin and AICAR significantly increased the expressions of *GFAP* and *BMP2* and repressed the expression of *MBP*; also, luteolin did not affect *TUBB3* expression, while AICAR increased its expression. And finally, while AICAR treatment increased the expressions of *STAT3*, *NOTCH1*, and *NOTCH3*, luteolin significantly decreased the expressions of these genes. Taken together, the repression of *MBP* expression and the increase of *BMP2* expression by luteolin and AICAR treatment suggest that both molecules induced the astrocytogenesis and repressed the oligodendrocytogenesis possibly *via* the Wnt-β catenin signaling pathway. The Wnt signaling, especially the canonical signaling (Wnt -β catenin), is crucial for NSC self-renewal and neurogenesis (Kasai et al., 2005; Wen et al., 2009); however, it also promotes astrocyte differentiation and suppresses oligodendroglial differentiation in a phase-dependent manner through BMP signal modulation (Kasai et al., 2005). Besides, the BMP signaling, in its turn, is reported to induce astrocytogenesis and repress oligodendrocyte genesis (Takouda et al., 2017). Among BMP family proteins, BMP2 signaling is reported to stimulate the astrocytic differentiation of NSCs *via* the initiation of the transcription of the astrogenic gene *GFAP* (Nakashima et al., 1999; Sloan and Barres, 2014; Takouda et al., 2017). Other studies reported that to stabilize the astrocyte phenotype, once *GFAP* is expressed, its transcription factor *STAT3*, together with the activated BMP signaling, triggers an auto-regulatory loop that reinforces itself and permits consolidation of the astrocyte phenotype (Wen et al., 2009). Upregulation of *BMP2* and *GFAP* observed in luteolin-treated hNSCs after 24 h of differentiation induction suggests that luteolin may have induced astrogenesis *via* the BMP2 signaling pathway. However, these results were contradictory with the significant decrease of the expression of the *STAT3* (at 24 h), the transcription factor of *GFAP*. Therefore, we opted to evaluate the expression of *STAT3*, its upstream regulator *BMP2*, and *GFAP* after 8 h of hNSCs differentiation induction by luteolin treatment. There was an increased expression of the *STAT3* in luteolin-treated hNSCs after 8 h, while *BMP2* and *GFAP* were not expressed. Since it was reported that GFAP expression depends on both *BMP2* signals and *STAT3* expression (Lee et al., 2015; Takouda et al., 2017), the non-expression of *GFAP* at 8 h in the present study would be attributed to the absence of *BMP2.* On the other hand, the increase of *BMP2* expression after 24 h of luteolin treatment may be, in part, permitting the activation of STAT3, which in turn permitted the expression of *GFAP* and, therefore, induced the differentiation of hNSCs into astrocytes. Moreover, the decrease of *STAT3* expression level after 24 h of luteolin treatment may highlight the activation of the autoregulatory loop that consolidates and stabilizes the astrocyte phenotype. Conjointly, the decreased expression of *NOTCH* receptors in our study (*NOTCH1* and *NOTCH3*) may highlight the inhibitory effect of luteolin treatment on hNSCs self-renewal process (Louvi and Artavanis-Tsakonas, 2006; Wen et al., 2009; Venkatesh et al., 2017). On the other hand, AICAR treatment decreased the expression of *STAT3* after 8 h of treatment; however, it increased the expression of, *GFAP* at 24 h, suggesting that AICAR did not enhance the astrogenesis process exclusively *via* STAT3 signaling. Besides, in contrast to luteolin treatment, AICAR treatment significantly increased the expression of *NOTCH3* at 24 h of treatment as well as increased the expression of *NOTCH1*, highlighting that AICAR probably induced the astrogenesis *via* Notch signaling. Moreover, the upregulation of *NOTCH1* and *NOTCH3* expressions may witness that AICAR treatment maintains the self-renewal properties of hNSCs (Nagao et al., 2007). Finally, the unchanged expression of *TUBB3* in luteolin-treated hNSCs, and the significant upregulation of this gene expression in AICAR-treated cells after 24 h treatment may highlight that contrary to AICAR, which show neurogenic potential, luteolin has a specific astrogenic effect on hNSCs.

**FIGURE 6:**
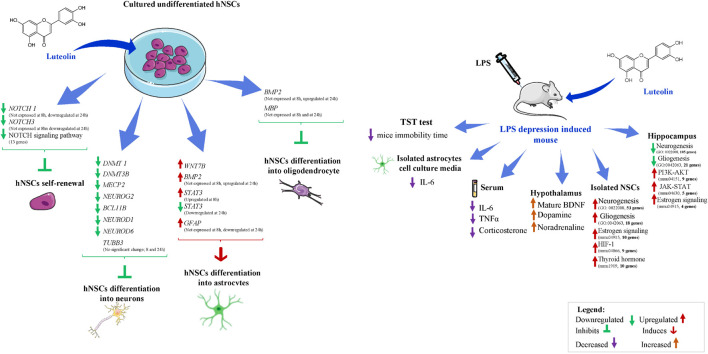
Schematic diagram of the effects of luteolin on human neural stem cells and on LPS-induced depression mice. Images are downloaded from www.smart.servier.com.

In summary, a comparison of the main molecular changes observed in both luteolin and AICAR-treated hNSCs allows us to conclude that the luteolin is a specific astrogenic enhancer molecule, while AICAR is an astrogenic and neurogenic enhancer as well as self-renewal maintainer of hNSCs. Moreover, these molecular changes may suggest that the astrogenic effect of luteolin on hNSCs was exclusively through the Wnt-β-catenin-BMP2-STAT3 pathway. In contrast, AICAR enhanced both astrogenic and neurogenic differentiation, as well as maintained self-renewal through both Notch and Wnt-β-catenin-BMP2-STAT3 signaling. Figure 6 summarizes the observed molecular effects of luteolin on hNSCs fate determination.

During recent years, an increasing amount of evidence has suggested the vast potential of NSCs in the area of translational medicine in debilitating neurodegenerative diseases. In this context, the use of natural compounds, such as polyphenols, are also being extensively studied for their potential stimulatory and synergistic effects on stem cells against neurodegenerative diseases (Tandon et al., 2018). There has been an emerging concept of astrocytes as mediators of polyphenol action in the CNS (Matias et al., 2016). Astrocytes are the most abundant type of cells in the brain and play an important role in nervous system integrity. Recent studies highlighted the putative roles of astrocytogenesis in both psychiatric including depression and in neurodegenerative disorders (Altshuler et al., 2010; Rajkowska and Stockmeier, 2013; Lee et al., 2015; Peng et al., 2015; Rial et al., 2016; Apple et al., 2017; Gonzales et al., 2017; Peng and Bonaguidi, 2018). It has been reported that under a loss of cerebral parenchymal integrity, astrocytes maintain homeostatic functions by removing excess glutamate in the synaptic cleft, promoting synaptogenesis, releasing neurotrophic factors, and regulating the blood flow during neuronal activity (Becerra-Calixto and Cardona-Gómez, 2017). It has also been reported that astrocyte homeostasis is tightly influenced by both the acute and chronic use of psychotropic drugs (Hertz et al., 2014). Thus, targeting the modulation of astrocytogenesis along with neurogenesis and/or oligodendrocytogenesis to re-establish the physiological CNS functions in the damaged brain, has been of great interest to treat the aforementioned pathologies. Therefore, our study offers a promising perspective on the beneficial use of luteolin in neuronal diseases.

To validate the modulatory effect of luteolin on astrocytogenesis observed *in vitro*, and to investigate its effect on neurogenesis and/or oligodendrocytogenesis to re-establish the physiological CNS functions in the damaged brain, we evaluated the effect of this flavone *in vivo* in mice animal model.

Although statistically non-significant, our results from the TST test, a test of antidepressant activity in mice where antidepressants decrease the time spent immobile without increasing general locomotor activity (Carr and Lucki, 2010), indicated that luteolin treatment slightly decreased the mice immobility time.

Findings from the quantification of astrocytes cells isolated from whole brains showed that LPS injection significantly reduced the number of astrocytes compared to that of LPS-induced depression mice treated with luteolin and control mice (*p* = *0.008*), however, luteolin treatment didn’t restore this cell loss. On the other hand, results from isolated mice astrocytes showed that secretion of IL-6 by astrocytes isolated from both the control group (PBS group mice) and the control group treated with luteolin (PBS group + L mice) was unchanged. Besides, this cytokine level was significantly increased in the cell culture media of astrocytes isolated from LPS treated mice, and it was significantly decreased in the cell culture media of astrocytes isolated from luteolin treated LPS-induced depression mice. These results highlighted the installation of the neuroinflammatory process in LPS treated mice that was neutralized by the luteolin treatment. In this context different studies, reported the secretion of IL-6 by astrocytes in different pathological conditions including major depression disease (Erta et al., 2012). Taken together the findings on these isolated cells highlight that luteolin treatment attenuated LPS-induced inflammatory responses in mice brain-derived astrocytes rather than increasing the number of astrocytes in mice brains.

Recently, it was shown that inflammation is involved in the pathogenesis of depression (Lee and Giuliani, 2019), and that some phytochemicals exert their anti-depressant effects through their anti-inflammatory effect (Wu et al., 2019). In the present study, the levels of Il-6, TNFα, and corticosterone were quantified in the sera of LPS induced depression mice. Results showed that LPS injection significantly increased the Il-6, TNFα, and corticosterone levels in mice sera, suggesting the establishment of the inflammatory stress. The oral administration of luteolin significantly reduced this increase depicting therefore the anti-inflammatory effect of luteolin.

Sabti et al. reported that antidepressants targeting the expression of serotonin and noradrenaline only present limitations and that accordingly antidepressant drugs targeting the dopaminergic system have been developed, whereas the antidepressants acting on serotonergic mechanisms lead to enhancement of BDNF levels in rodents (Sabti et al., 2019). Hence, we assessed the mature BDNF levels, noradrenaline and dopamine levels in mice hypothalamus and serotonin level in mice cortex. The results showed that: mature BDNF levels were significantly increased in the hypothalamus of PBS and LPS group when they are treated with Luteolin, noradrenaline levels were significantly increased in the hypothalamus of LPS group mice and it was significantly decreased when treated with luteolin, and dopamine levels were significantly decreased in the hypothalamus of LPS group mice and significantly increased when treated with luteolin.

Taken together, our results show that luteolin treatment may exert an antidepressant effect in the LPS induced depression mice by decreasing the IL-6 production by astrocytes, decreasing the serum IL-6, TNFα, and corticosterone and by increasing the levels of mature BDNF, dopamine, and noradrenaline levels in the hypothalamus.

The evaluation of the transcriptome in the isolated mice NSCs and mice hippocampi showed that LPS injection significantly downregulated several neuronal and glial biological processes including the neurogenesis (GO: 0022008) and gliogenesis (GO:0042063) that were mostly upregulated with luteolin treatment in mice NSCs and not in hippocampi. In NSCs, except the *Camk2a* gene, all the downregulated genes by LPS injection were upregulated after luteolin treatment pointing out the restoring effect exerted by luteolin treatment on mice NSCs to overcome the damage related to neurogenesis and astrogenesis processes caused by LPS injection. *Camk2a* gene encodes the subunit alpha of CaMKII protein. This protein isoform to is known to be mainly expressed in the brain neurons and it is well established that its abnormal functioning is linked to the pathophysiology of depression. So far most studies associated the decrease in CaMKII alpha levels to the depressive like phenotype in animal models and highlighted the role of antidepressants to increase this protein expression (Sałaciak et al., 2021). In their review paper Salaciak and colleagues discussed the effect of CaMKII in antidepressant-like effect installation mentioning that this activity requires the activation of both CaMKIIα and CaMIV together with the inactivation of CaMKIIβ. Ahmed and colleagues associated the inhibition of CaMKIIα expression to an anxiolytic effect (Ahmed et al., 2017).

Our *in vivo* transcriptomic study showed also that LPS injection enriched different KEGG signaling pathways by downregulation in mice NSCs with the estrogen signaling (mmu04915) being the most enriched signaling pathway (*p* = 9.87E-05). Luteolin treatment enriched by upregulation, the estrogen signaling (mmu04915, *p* = 0.0093), the HIF-1 (mmu04066) and the thyroid hormone (mmu1919). In the NSCs of normal mice group, it enriched the Wnt signaling by upregulation.

Recently, the role of estrogen signaling has been highlighted with studies reporting its neuroprotective effects on the brain by sheltering it from inflammation and stress (Azcoitia et al., 2019; Hwang et al., 2021), and investigations in animal models of psychiatric disorders and in patients, revealed that this signaling is disturbed and associated with cognitive deficits and also with the manifestations of symptoms that could be reversed with treatments targeting estrogen-signaling pathways (Hwang et al., 2021). To add more Li et al. showed that hypoxia-inducible factor-1 (HIF-1) pathway is a promising target for the treatment of depression (Li et al., 2020). Indeed, they reported that HIF-1 may produce beneficial effects on depression, as it targets genes that have been shown to elicit antidepressant effects in animal models (erythropoietin (EPO) and vascular endothelial growth factor (VEGF) genes (Li et al., 2020).

On the other hand, thyroid hormone signaling, a key modulator of neuropsychiatric disorders including depression, is known as a regulator of the developmental program in the brain. The thyroid hormone action targets diverse cellular processes such as progenitor turnover, cell survival and differentiation, cellular homeostasis, and metabolic regulation. In mice embryonic neural stem cells, it promotes neuronal differentiation by inhibiting STAT3 signaling through the thyroid receptor α1, and inhibits astrocytic differentiation, in rodents Thyroid Hormone signaling activates mitochondrial metabolism during NSC commitment to neuronal precursor cells, and in humans, it regulates hippocampal neurogenesis dependent behaviors such as mood-related behaviors (Ming and Song, 2011; Chen et al., 2012; Kapoor et al., 2015; Miller and Hen, 2015; Gothié et al., 2020).

As previously mentioned, the Wnt signaling plays a curcial role for NSC self-renewal and neurogenesis (Kasai et al., 2005; Wen et al., 2009); and it promotes astrocyte differentiation and suppresses oligodendroglial differentiation in a phase-dependent manner through BMP signal modulation (Kasai et al., 2005).

Considering these findings, our results point out an anti-depressant effect of luteolin treatment exerted especially *via* the modulation of the estrogen, HIF-1 and thyroid hormone signaling pathways in the NSCs of mice. Equally these findings highlight the modulatory effect exerted by luteolin on signaling pathways involved in NSCs fate determination namely the Wnt signaling.

In the hippocampus of LPS induced depression model mice, the luteolin treatment enriched the GABAergic synapse by downregulation, and the PI3K-AKT (mmu04151), the JAK-STAT (mmu04630), and the estrogen signaling (mmu04915) by upregulation.

Recently, PI3K/Akt/GSK-3β/mTOR signaling pathway has been associated to neurobiology of depression and seems to be modulated by some pharmacological antidepressant strategies. Ludka et al. (2016) have shown a behavioral anti-depressant effect of Atorvastatin that was supported by neurochemical observations revealed by an increase of the immunocontent of the phosphorylated isoforms of Akt, GSK-3β and mTOR in the hippocampus of mice (Ludka et al., 2016).

In addition to its aforementioned role in the modulation of GFAP gene expression, JAK/STAT pathway is involved in mediating several functions of the central nervous system, including neurogenesis, synaptic plasticity, gliogenesis, and microglial activation, and all of which have been implicated in the pathophysiology of mood disorders. Moreover, there is also direct evidence from studies in populations with depressive disorders, suggesting that JAK/STAT pathways may be involved in the pathophysiology of depression and the antidepressant actions of current treatments have been shown to be mediated by JAK/STAT-dependent mechanisms (Wen et al., 2009; Lee et al., 2015; Shariq et al., 2019). Figure 6 summarizes the observed molecular effects of luteolin LPS-induced depression mice.

Thus, along with the results obtain in our *in vitro* study using hNSCs, the *in vivo* study confirms the potential astrogenic effect of luteolin in the defected astrogenesis model and highlights its modulatory effect on WNT-JAK-STAT signaling. This *in vivo* study highlighted also the modulatory effect of luteolin on different signaling pathways involved in the pathophysiology of depression. So far, the anti-depressant effect of luteolin treatment *via* Suppressing Endoplasmic Reticulum Stress was reported (Ishisaka et al., 2011), however to the best of our knowledge, this is the first study to show: that luteolin treatment might alter some signaling pathways associated with depression *via* the modulation of NSCs fate determination and that this flavone had are restoring effect on NSCs signaling to overcome the damage caused by LPS injection.

Given the non-static nature of NSCs, the effects of its source and its derivation method that may significantly affect its differentiation potential into specific cell types, the disrupt of commercialization of the hNSCs used in our study (StemPro^TM^ neural stem cells, Cat. no. A15654) limited the study of the protein expression of genes evaluated by RT-qPCR as well as the study of the composition of the NSC population including the percentage of SOX2 and PAX6 expression that would be informative and the percentage of mature neurons and TUBB3 percentage within the NSC population. The results from this *in vitro* study, therefore, require a future replication with other type of hNSCs.

## Conclusion

In summary, our *in vitro* study in hNSCs showed that luteolin significantly increased the expression of *GFAP* and the number of GFAP+ cells as well as altered the WNT-BMP2-STAT3 pathways suggesting its potential for astrocytogenesis. Our *in vivo* findings showed luteolin significantly attenuated LPS-induced neuroinflammation by decreasing the IL-6 production in mice brain-derived astrocytes, reducing the serum IL-6, TNFα and corticosterone levels, and increasing the mature BDNF, dopamine, and noradrenaline levels in the hypothalamus. Whole-genome transcriptome analysis suggests that luteolin treatment may restore LPS-induced alterations in biological functions related to neurogenesis and astrocytogenesis in mice hypothalamus and brain-derived NSCs. Although the antidepressant behavioral effects of luteolin did not reach statistical significance, a number of signaling pathways involved in the pathophysiology of depression were modulated by luteolin treatment. Our study is the first to report astrocytogenic potential of luteolin and thus highlighting its possible therapeutic benefits in neuroinflammatory and neurodegenerative diseases. However, future studies are required to confirm its molecular mechanism of action.

## Data Availability Statement

All data generated or analyzed during this study are included in this published article and its supplementary information files. Microarray data are deposited in the Gene Expression Omnibus (GEO) under Accession Numbers: GSE148160 (https://www.ncbi.nlm.nih.gov/geo/query/acc.cgi?acc=GSE148160), GSE181285 (https://www.ncbi.nlm.nih.gov/geo/query/acc.cgi?acc=GSE181285), and GSE181522 (https://www.ncbi.nlm.nih.gov/geo/query/acc.cgi?acc=GSE181522).

## Ethics Statement

The animal study was reviewed and approved by the Animal Ethics Committee of the University of Tsukuba, Japan.

## Author Contributions

MA contributed to conceptualization, methodology, investigation, formal analysis, visualization, software, and writing – original draft. FF contributed to formal analysis, visualization, software, validation, and writing – review and editing. KS contributed to methodology, investigation, and supervision. HI contributed to conceptualization, resources, supervision, project administration, and funding acquisition. All authors read and approved the final manuscript.

## Conflict of Interest

The authors declare that the research was conducted in the absence of any commercial or financial relationships that could be construed as a potential conflict of interest.

## Publisher’s Note

All claims expressed in this article are solely those of the authors and do not necessarily represent those of their affiliated organizations, or those of the publisher, the editors and the reviewers. Any product that may be evaluated in this article, or claim that may be made by its manufacturer, is not guaranteed or endorsed by the publisher.
