# Supplementary material for: Luteolin Modulates Neural Stem Cells Fate Determination: In vitro Study on Human Neural Stem Cells, and in vivo Study on LPS-Induced Depression Mice Model
Source: Front Cell Dev Biol. 2021 Nov 1;9:753279. doi: 10.3389/fcell.2021.753279 (PMC8591246; doi:10.3389/fcell.2021.753279)
Supplement: Supplementary file 1 [file Image_1.pdf]

Supplementary Figures

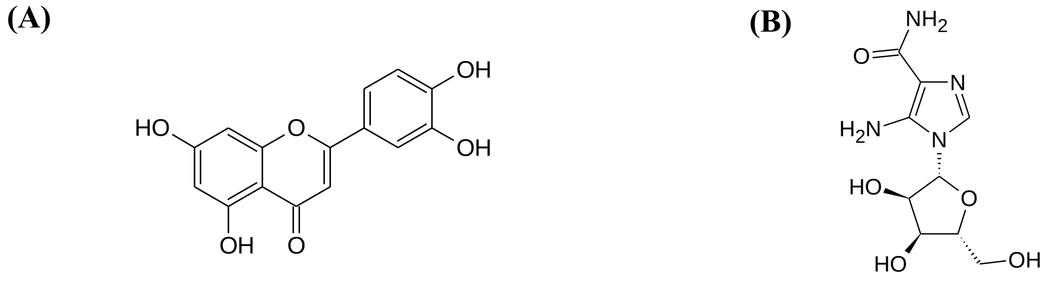

Supplementary Figure 1. Chemical structures. (A) Luteolin, (B) AICAR

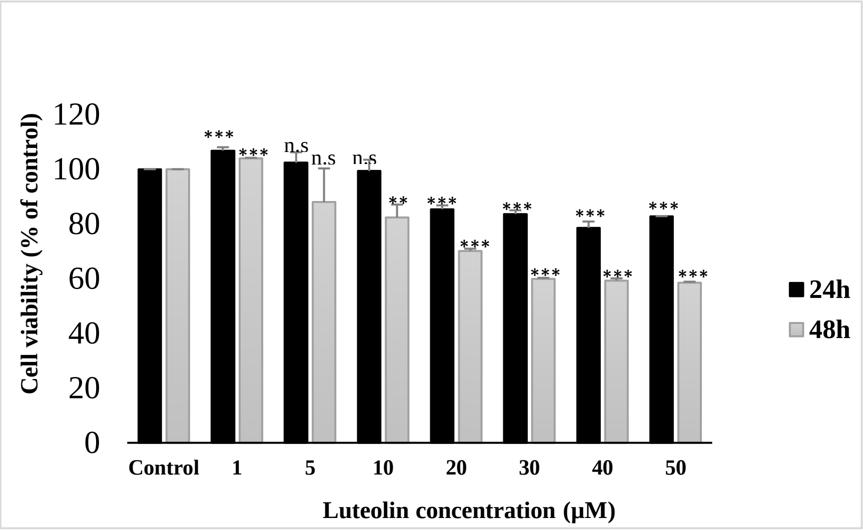

Supplementary Figure 2. 1µM luteolin slightly increased cell viability of SH-SY5Y cells after 24 h treatment. (A) Cell viability of SH-SY5Y cells treated with luteolin for 24 h and 48 h. Cell iability as determined using MTT assay. Data are expressed as the mean ± SD of four independent experiments. ***$p < 0.001$, **$p \leq 0.01$ and *$p<0.05$ (Student's t-test).

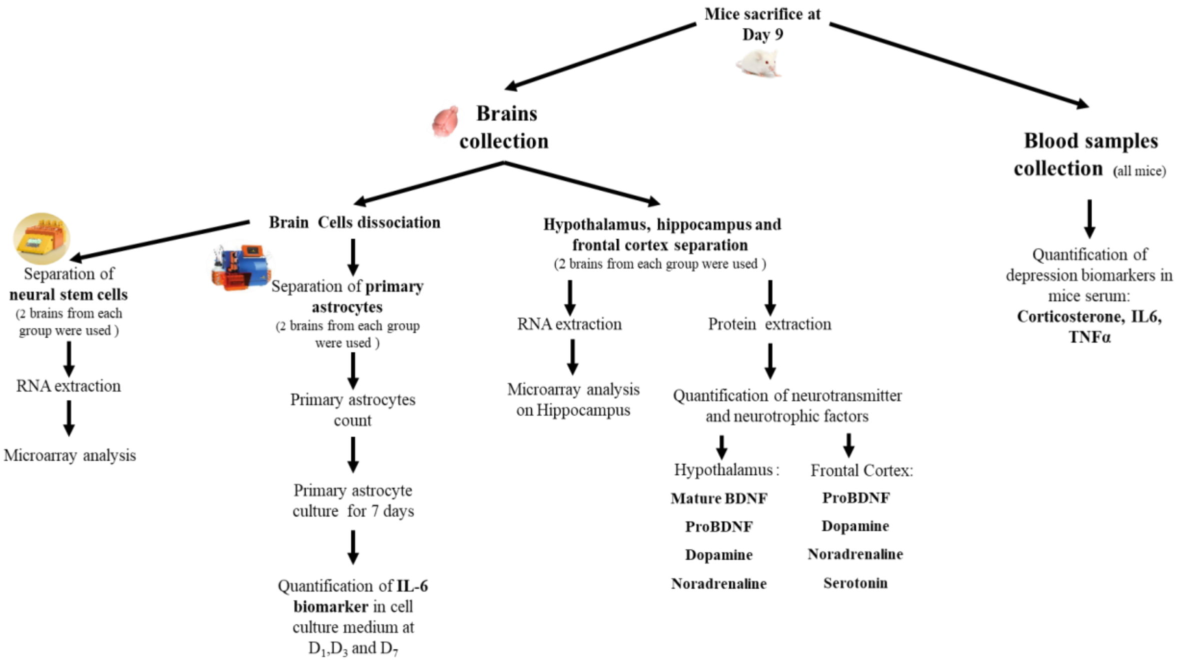

Supplementary Figure 3. Summary of animal experiments

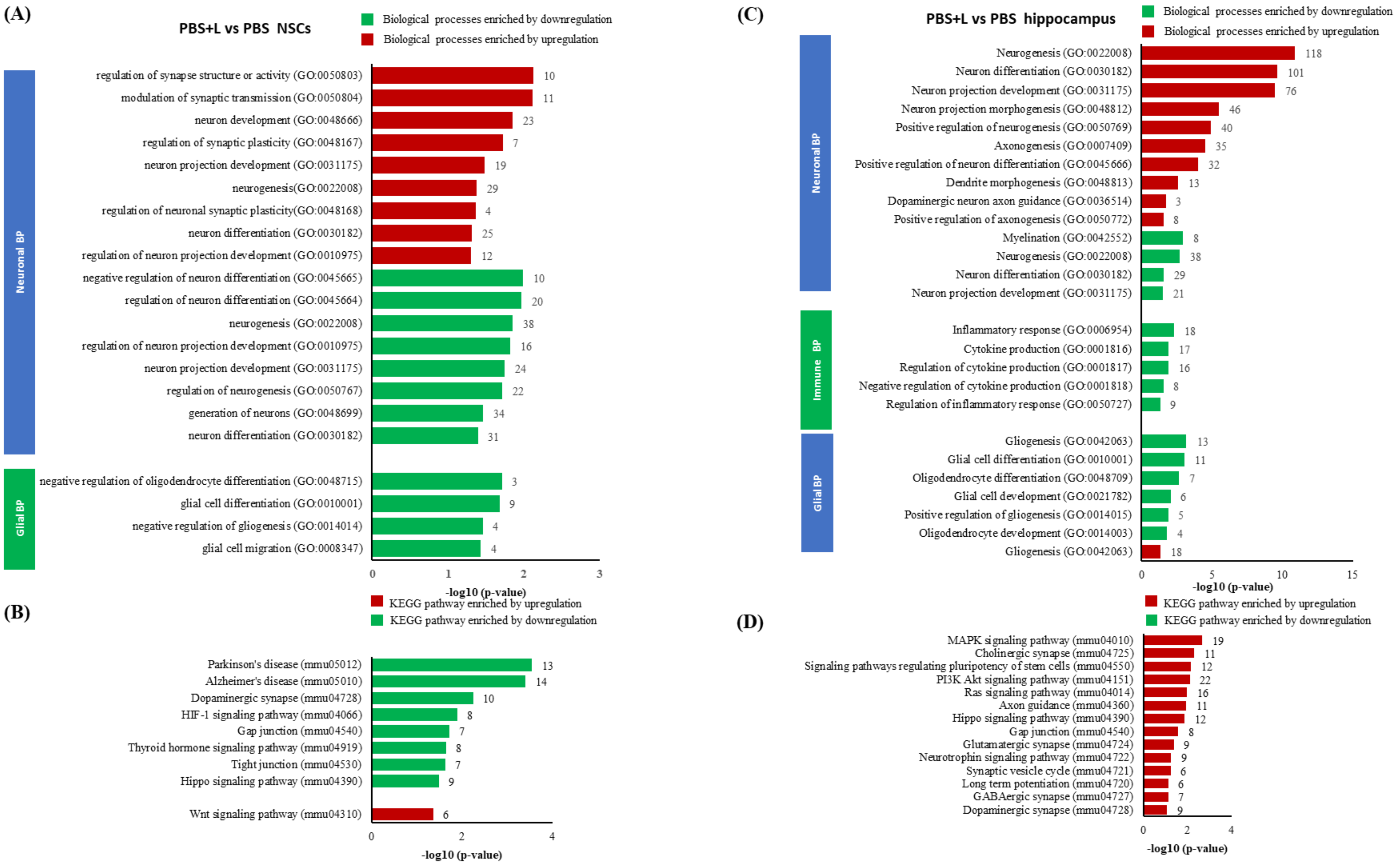

Supplementary Figure 4. Luteolin treatment significantly regulated gene expression in NSCs and hippocampus of normal mice. (A, B) Differentially regulated biological processes (BP) and top significantly enriched KEGG signaling pathways in NSCs of normal mice treated with luteolin (PBS+L) compared to untreated normal mice (PBS). **(C, D)** Differentially regulated biological processes (BP) and top significantly enriched KEGG signaling pathways in hippocampus of normal mice treated with luteolin (PBS+L) compared to untreated normal mice (PBS).
