# Supplementary material for: Luteolin Modulates Neural Stem Cells Fate Determination: In vitro Study on Human Neural Stem Cells, and in vivo Study on LPS-Induced Depression Mice Model
Source: Front Cell Dev Biol. 2021 Nov 1;9:753279. doi: 10.3389/fcell.2021.753279 (PMC8591246; doi:10.3389/fcell.2021.753279)
Supplement: Supplementary file 2 [file Table_1.pdf]

# Supplementary tables

## Supplementary table 1. Top 10 upregulated genes after 24 h differentiation induction of human Neural Stem cells by 1 µM luteolin

| Gene symbol | Gene title | Fold change | *p*-value | Gene function* |
|---|---|---|---|---|
| **HCLS1** | Hematopoietic cell-specific Lyn substrate 1 | 1.85 | 0.0486 | Plays a role in antigen receptor signaling for both clonal expansion and deletion in lymphoid cells. May also be involved in the regulation of gene expression. |
| **FOXJ2** | Forkhead box J2 | 1.84 | 0.0422 | The *FOXP2* gene provides instructions for making a protein called forkhead box P2. The forkhead box P2 protein is active in several tissues, including the brain, both before and after birth. Studies suggest that it plays important roles in brain development, including the growth of nerve cells (neurons) and the transmission of signals between them. It is also involved in synaptic plasticity, which is the ability of connections between neurons (synapses) to change and adapt to experience over time. Synaptic plasticity is necessary for learning and memory. |
| **SCNN1G** | Sodium channel, non-voltage gated 1 gamma subunit | 1.83 | 0.0056 | The *SCNN1G* gene provides instructions for making one piece, the gamma subunit, of a protein complex called the epithelial sodium channel (ENaC). The channel is composed of alpha, beta, and gamma subunits, each of which is produced from a different gene. These channels are found at the surface of certain cells called epithelial cells in many tissues of the body, including the kidneys, lungs, and sweat glands. The ENaC channel transports sodium into cells. |
| **RNF216** | Ring finger protein 216 | 1.8 | 0.0117 | The *RNF216* gene provides instructions for making a protein that plays a role in the ubiquitin-proteasome system. Specifically, this protein functions as an E3 ubiquitin ligase. The RNF216 protein tags proteins involved in an early immune response called inflammation to help control the response. RNF216 also regulates the amount of a protein in nerve cells (neurons) called Arc, which plays a role in a process called synaptic plasticity. Synaptic plasticity is the ability of the connections between neurons (synapses) to change and adapt over time in response to experience. This process is critical for learning and memory. It is likely that thn RNF216 protein also regulates proteins involved in other body processes, although these proteins have not been identified. |
| **PPIAP30** | Peptidylprolyl isomerase A (cyclophilin A) pseudogene 30 | 1.75 | 0.0179 | This gene encodes a member of the peptidyl-prolyl cis-trans isomerase (PPIase) family. PPIases catalyze the cis-trans isomerization of proline imidic peptide bonds in oligopeptides and accelerate the folding of proteins. The encoded protein is a cyclosporin binding-protein and may play a role in cyclosporin A-mediated immunosuppression. The protein can also interact with several HIV proteins, including p55 gag, Vpr, and capsid protein, and has been shown to be necessary for the formation of infectious HIV virions. |
| **MAP9** | Microtubule-associated protein 9 | 1.69 | 0.0167 | This gene encodes the microtubule-associated protein that is required for spindle function, mitotic progression, and cytokinesis |
| **DROSHA** | Drosha, ribonuclease type III | 1.66 | 0.0051 | This gene encodes a ribonuclease (RNase) III double-stranded RNA-specific ribonuclease and subunit of the microprocessor protein complex, which catalyzes the initial processing step of microRNA (miRNA) synthesis. The encoded protein cleaves the stem loop structure from the primary microRNA (pri-miRNA) in the nucleus, yielding the precursor miRNA (pre-miRNA), which is then exported to the cytoplasm for further processing. |
| **SP3** | Sp3 transcription factor | 1.66 | 0.0415 | This gene belongs to a family of Sp1 related genes that encode transcription factors that regulate transcription by binding to consensus GC- and GT-box regulatory elements in target genes. This protein contains a zinc finger DNA-binding domain and several transactivation domains, and has been reported to function as a bifunctional transcription factor that either stimulates or represses the transcription of numerous genes. |
| **NELFA** | Negative elongation factor complex member A | 1.64 | 0.0034 | The encoded protein is found to be capable of reacting with HLA-A2-restricted and tumor-specific cytotoxic T lymphocytes, suggesting a target for use in specific immunotherapy for a large number of cancer patients |
| **GLUD1** | Glutamate dehydrogenase 1 | 1.63 | 0.002 | This gene encodes glutamate dehydrogenase, which is a mitochondrial matrix enzyme that catalyzes the oxidative deamination of glutamate to alpha-ketoglutarate and ammonia. |

*Gene function as reported in NCBI ( **https://www.ncbi.nlm.nih.gov/guide/howto/find-func-gene/** )

## Supplementary table 2. Top 10 downregulated genes after 24 h differentiation induction of human Neural Stem cells by1μM luteolin

| Gene symbol | Gene title | Fold change | *p*-value | Gene function* |
|---|---|---|---|---|
| **LSM12** | LSM12 homolog | -1.87 | 0.0004 | LSM12 acts as a molecular adaptor for the recruitment of TWENTY-FOUR (TYF) to ATX2. The ATX2-LSM12- TYF complex thereby stimulates TYF-dependent translation of the rate-limiting clock gene period (per) to maintain 24 hr periodicity in circadian behaviors |
| **MAP4K2** | Mitogen-activated protein kinase kinase kinase kinase 2 | -1.87 | 0.0153 | The protein encoded by this gene is a member of the serine/threonine protein kinase family. This kinase can be activated by TNF-alpha, and has been shown to specifically activate MAP kinases. This kinase is also found to interact with TNF receptor-associated factor 2 (TRAF2), which is involved in the activation of MAP3K1/MEKK1. |
| **IL1RL1** | Interleukin 1 receptor-like 1 | -1.83 | 0.0141 | The protein encoded by this gene is a member of the interleukin 1 receptor family. Studies of the similar gene in mouse suggested that this receptor can be induced by proinflammatory stimuli, and may be involved in the function of helper T cells. |
| **TAF15** | TATA box binding protein associated factor 15 | -1.82 | 0.0224 | This gene encodes a member of the TET family of RNA-binding proteins. The encoded protein plays a role in RNA polymerase II gene transcription as a component of a distinct subset of multi-subunit transcription initiation factor TFIID complexes. Translocations involving this gene play a role in acute leukemia and extraskeletal myxoid chondrosarcoma, and mutations in this gene may play a role in amyotrophic lateral sclerosis |
| **EXOC7** | Exocyst complex component 7 | -1.78 | 0.0063 | The protein encoded by this gene is a component of the exocyst complex. The exocyst complex plays a critical role in vesicular trafficking and the secretory pathway by targeting post-Golgi vesicles to the plasma membrane. The encoded protein is required for assembly of the exocyst complex and docking of the complex to the plasma membrane. The encoded protein may also play a role in pre-mRNA splicing through interactions with pre-mRNA-processing factor 19. |
| **SFSWAP** | Splicing factor, suppressor of white-apricot family | -1.77 | 0.0001 | This gene encodes a human homolog of Drosophila splicing regulatory protein. This gene autoregulates its expression by control of splicing of its first two introns. In addition, it also regulates the splicing of fibronectin and CD45 genes |
| **EHBP1** | EH domain binding protein 1 | -1.77 | 0.0081 | This gene encodes an Eps15 homology domain binding protein. The encoded protein may play a role in endocytic trafficking. A single nucleotide polymorphism in this gene is associated with an aggressive form of prostate cancer. |
| **SNORA54** | Small nucleolar RNA, H/ACA box 54 | -1.74 | 0.0169 | This is a non-coding RNA (ncRNA) molecule which functions in the biogenesis (modification) of other small nuclear RNAs |
| **KIF26B** | Kinesin family member 26B | -1.73 | 0.0067 | The protein encoded by this gene is an intracellular motor protein thought to transport organelles along microtubules. The encoded protein is required for kidney development. Elevated levels of this protein have been found in some breast and colorectal cancers. |
| **CACTIN** | Cactin, spliceosome C complex subunit | -1.72 | 0.0213 | Acts as negative regulator of Toll-like receptor and interferon-regulatory factor (IRF) signaling pathways. Contributes to the regulation of transcriptional activation of NF-kappa-B target genes in response to endogenous proinflammatory stimuli. |

*Gene function as reported in NCBI ( https://www.ncbi.nlm.nih.gov/guide/howto/find-func-gene/ )

## Supplementary table 3. Genes involved in astrocytes differentiation (GO:0048708)

| ID | Gene Chip Array | Transcript ID (Array Design) | Gene Title | Gene Symbol | Luteolin Avg (log2) | Control Avg (log2) | Fold Change |
|---|---|---|---|---|---|---|---|
| **11728643_s_at** | Human Genome HG-U219 Array | Hs.433512.1 | ARP3 actin-related protein 3 homolog (yeast) | ACTR3 | -0.12 | -0.33 | 1.16 |
| **11728973_at** | Human Genome HG-U219 Array | Hs.57971.1 | hes family bHLH transcription factor 5 | HES5 | -0.38 | -0.42 | 1.03 |
| **11727162_x_at** | Human Genome HG-U219 Array | Hs.257970.1 | nuclear factor I/X (CCAAT-binding transcription factor) | NFIX | -0.17 | -0.54 | 1.29 |
| **11727160_at** | Human Genome HG-U219 Array | Hs.257970.1 | nuclear factor I/X (CCAAT-binding transcription factor) | NFIX | 0.04 | -0.31 | 1.27 |
| **11731863_at** | Human Genome HG-U219 Array | Hs.516922.1 | NK2 homeobox 2 | NKX2-2 | -0.32 | -0.36 | 1.02 |
| **11722466_s_at** | Human Genome HG-U219 Array | Hs.270303.1 | paired box 6 | PAX6 | -0.39 | -0.55 | 1.12 |
| **11757957_s_at** | Human Genome HG-U219 Array | Hs.712586.1 | SRY box 6 | SOX6 | -0.18 | -0.42 | 1.18 |

## Supplementary table 4. Genes involved in positive regulation of astrocytes differentiation (GO:0048711)

| ID | Gene Chip Array | Transcript ID (Array Design) | Gene Title | Gene Symbol | Luteolin Avg (log2) | Control Avg (log2) | Fold Change |
|---|---|---|---|---|---|---|---|
| **11728643_s_at** | Human Genome HG-U219 Array | Hs.433512.1 | ARP3 actin-related protein 3 homolog (yeast) | ACTR3 | -0.12 | -0.33 | 1.16 |
| **11746895_a_at** | Human Genome HG-U219 Array | Hs.193163.10 | bridging integrator 1 | BIN1 | -0.01 | -0.19 | 1.14 |
| **11743498_at** | Human Genome HG-U219 Array | Hs.73853.1 | bone morphogenetic protein 2 | BMP2 | -0.44 | -0.28 | -1.11 |
| **11749378_a_at** | Human Genome HG-U219 Array | Hs.502977.2 | cardiotrophin-like cytokine factor 1 | CLCF1 | -0.25 | -0.43 | 1.13 |
| **11760345_a_at** | Human Genome HG-U219 Array | Hs.250666.2 | hes family bHLH transcription factor 1 | HES1 | 0.2 | -0.13 | 1.26 |
| **11746878_s_at** | Human Genome HG-U219 Array | Hs.591670.1 | inhibitor of DNA binding 2, dominant negative helix-loop-helix protein | ID2 | -0.01 | -0.2 | 1.14 |
| **11719916_at** | Human Genome HG-U219 Array | Hs.126256.1 | interleukin 1 beta | IL1B | -0.31 | -0.48 | 1.13 |
| **11730757_a_at** | Human Genome HG-U219 Array | Hs.532082.1 | interleukin 6 signal transducer | IL6ST | -0.23 | -0.44 | 1.15 |
| **11726497_at** | Human Genome HG-U219 Array | Hs.2250.1 | leukemia inhibitory factor | LIF | -0.4 | -0.25 | -1.11 |
| **11734656_a_at** | Human Genome HG-U219 Array | Hs.643440.2 | myelin associated glycoprotein | MAG | -0.12 | -0.03 | -1.07 |
| **11718261_at** | Human Genome HG-U219 Array | Hs.495473.1 | notch 1 | NOTCH1 | 0.1 | 0.2 | -1.07 |
| **11716736_at** | Human Genome HG-U219 Array | Hs.502705.1 | pre-mRNA processing factor 19 | PRPF19 | -0.38 | -0.39 | 1.01 |
| **11758598_s_at** | Human Genome HG-U219 Array | Hs.667987.1 | serpin peptidase inhibitor, clade E (nexin, plasminogen activator inhibitor type 1), member 2 | SERPINE2 | 0.05 | -0.24 | 1.22 |

## Supplementary table 5. Genes involved in negative regulation of astrocytes differentiation (GO:0048712)

| ID | Gene Chip Array | Transcript ID (Array Design) | Gene Title | Gene Symbol | Luteolin Avg (log2) | Control Avg (log2) | Fold Change |
|---|---|---|---|---|---|---|---|
| **11728643_s_at** | Human Genome HG-U219 Array | Hs.433512.1 | ARP3 actin-related protein 3 homolog (yeast) | ACTR3 | -0.12 | -0.33 | 1.16 |
| **11717801_a_at** | Human Genome HG-U219 Array | Hs.9754.1 | activating transcription factor 5 | ATF5 | -0.42 | -0.42 | 1 |
| **11759050_at** | Human Genome HG-U219 Array | Hs.477370.1 | Dab, reelin signal transducer, homolog 1 (Drosophila) | DAB1 | -0.49 | 0.05 | -1.46 |
| **11743768_a_at** | Human Genome HG-U219 Array | Hs.127792.2 | delta-like 3 (Drosophila) | DLL3 | -0.03 | 0.2 | -1.17 |
| **11723004_x_at** | Human Genome HG-U219 Array | Hs.655207.1 | coagulation factor II (thrombin) | F2 | -0.28 | -0.14 | -1.1 |
| **11755786_a_at** | Human Genome HG-U219 Array | Hs.1420.4 | fibroblast growth factor receptor 3 | FGFR3 | -0.17 | -0.14 | -1.02 |
| **11728973_at** | Human Genome HG-U219 Array | Hs.57971.1 | hes family bHLH transcription factor 5 | HES5 | -0.38 | -0.42 | 1.03 |
| **11734711_at** | Human Genome HG-U219 Array | Hs.132049.1 | G protein-coupled receptor 37 like 1 | GPR37L1 | 0.27 | 0.14 | 1.1 |
| **11762061_at** | Human Genome HG-U219 Array | Hs.505924.4 | high mobility group AT-hook 2 | HMGA2 | -0.11 | 0.08 | -1.14 |
| **11721689_x_at** | Human Genome HG-U219 Array | Hs.519601.1 | inhibitor of DNA binding 4, dominant negative helix-loop-helix protein | ID4 | -0.37 | -0.08 | -1.23 |
| **11726730_a_at** | Human Genome HG-U219 Array | Hs.155983.1 | lysine (K)-specific demethylase 4A | KDM4A | -0.48 | -0.5 | 1.01 |
| **11755323_x_at** | Human Genome HG-U219 Array | Hs.405610.11 | methyl-CpG binding domain protein 1 | MBD1 | -0.13 | 0.01 | -1.11 |
| **11722683_a_at** | Human Genome HG-U219 Array | Hs.200716.1 | methyl-CpG binding protein 2 | MECP2 | -0.39 | -0.24 | -1.11 |
| **11719870_s_at** | Human Genome HG-U219 Array | Hs.25960.1 | v-myc avian myelocytomatosis viral oncogene neuroblastoma derived homolog | MYCN | -0.1 | -0.08 | -1.02 |
| **11763504_at** | Human Genome HG-U219 Array | Hs.113577.8 | neurofibromin 1 | NF1 | -0.18 | 0.12 | -1.24 |
| **11740638_at** | Human Genome HG-U219 Array | Hs.248201.1 | noggin | NOG | -0.37 | -0.47 | 1.07 |
| **11733365_a_at** | Human Genome HG-U219 Array | Hs.157688.1 | nuclear receptor subfamily 2, group E, member 1 | NR2E1 | -0.39 | -0.44 | 1.04 |
| **11727685_at** | Human Genome HG-U219 Array | Hs.410969.1 | neurotrophic tyrosine kinase, receptor, type 3 | NTRK3 | -0.23 | 0.03 | -1.2 |

**Supplementary table 6. Top 10 upregulated genes in the isolated NSCs of LPS-induced depression mice treated with luteolin compared to untreated LPS-induced depression mice.**

| Gene symbol | Gene title | Fold change | *p*-value | Gene function* |
|---|---|---|---|---|
| **Hba-a1; Hba-a2** | hemoglobin alpha, adult chain 1; hemoglobin alpha, adult chain 2 | 7.29 | 4.12E-05 | Involved in oxygen transport from the lung to the various peripheral tissues. |
| **Hbb-b1; Hbb-b2; Hbb-bs; Hbb-bt** | hemoglobin, beta adult major chain; hemoglobin, beta adult minor chain; hemoglobin, beta adult s chain; hemoglobin, beta adult t chain | 7.22 | 1.24E-05 | Involved in oxygen transport from the lung to the various peripheral tissues. |
| **Clu** | clusterin | 5.27 | 1.77E-07 | Functions as extracellular chaperone that prevents aggregation of non-native proteins. Prevents stress-induced aggregation of blood plasma proteins. Inhibits formation of amyloid fibrils by APP, APOC2, B2M, CALCA, CSN3, SNCA and aggregation-prone LYZ variants. When secreted, protects cells against apoptosis and against cytolysis by complement. Modulates NF-kappa-B transcriptional activity. Following stress, promotes apoptosis. Inhibits apoptosis when associated with the mitochondrial membrane by interference with BAX-dependent release of cytochrome c into the cytoplasm. Following ER stress, suppresses stress-induced apoptosis by stabilizing mitochondrial membrane integrity through interaction with HSPA5. When secreted, acts as an important modulator during neuronal differentiation through interaction with STMN3. Plays a role in the clearance of immune complexes that arise during cell injury. |
| **Ly6a** | lymphocyte antigen 6 complex, locus A | 4.78 | 9.28E-08 | Involved in T cell activation |
| **Pltp** | phospholipid transfer protein | 4.55 | 2.28E-05 | Mediates the transfer of phospholipids and free cholesterol from triglyceride-rich lipoproteins into high-density lipoproteins as well as the exchange of phospholipids between triglyceride-rich lipoproteins themselves<br>Facilitates the transfer of a spectrum of different lipid molecules, including sphingomyelin, phosphatidylcholine, phosphatidylinositol, phosphatidylglycerol, and phosphatidyl ethanolamine. Plays an important role in HDL remodeling which involves modulating the size and composition of HDL. Also plays a key role in the uptake of cholesterol from peripheral cells and tissues that is subsequently transported to the liver for degradation and excretion. Two distinct forms of PLTP exist in plasma: an active form that can transfer phosphatidylcholine from phospholipid vesicles to HDL, and an inactive form that lacks this capability |
| **Tuba1a** | tubulin, alpha 1A | 4.27 | 7.67E-08 | Tubulin is the major constituent of microtubules. It binds two moles of GTP, one at an exchangeable site on the beta chain and one at a non-exchangeable site on the alpha chain. |
| **Gm1821; Ubb** | ubiquitin pseudogene; ubiquitin B | 4.04 | 1.52E-05 | It has distinct roles, such as in activation of protein kinases, and in signaling |
| **B2m** | beta-2 microglobulin | 4 | 1.21E-08 | Component of the class I major histocompatibility complex (MHC). Involved in the presentation of peptide antigens to the immune system. |
| **Pik3cd** | phosphatidylinositol 3-kinase catalytic delta polypeptide | 3.81 | 0.0009 | Mediates immune responses. Plays a role in B-cell development, proliferation, migration, and function. Required for B-cell receptor (BCR) signaling. Mediates B-cell proliferation response to anti-IgM, anti-CD40 and IL4 stimulation. Promotes cytokine production in response to TLR4 and TLR9. Involved in the antigen presentation function of B-cells. Required for proliferation, and signaling. Involved in stem cell factor (SCF)-mediated proliferation, adhesion and migration. |
| **Bsg** | basigin | 3.64 | 7.13E-06 | Involved in neural retina development; photoreceptor cell maintenance; and spermatogenesis. Localizes to several cellular components, including acrosomal membrane; photoreceptor inner segment; and photoreceptor outer segment. Is expressed in several structures, including brain and sensory organ. |

*Gene function as reported in NCBI ( **https://www.ncbi.nlm.nih.gov/guide/howto/find-func-gene/** )

## Supplementary table 7. Top 10 downregulated genes in the isolated NSCs of LPS-induced depression mice treated with luteolin compared to untreated LPS-induced depression mice.

| Gene symbol | Gene title | Fold change | *p*-value | Gene function* |
|---|---|---|---|---|
| **Ltf** | lactotransferrin | -2.12 | 0.001 | Exhibits peptidase activity. Predicted to be involved in several processes, including defense response to other organism; negative regulation of viral process; and regulation of signal transduction. Localizes to cytoplasm. Is expressed in several structures, including early conceptus; epithelium; female reproductive system; immune system; and liver. |
| **Lyz2** | lysozyme 2 | -2.1 | 0.0103 | Exhibits lysozyme activity. Involved in defense response to Gram-negative bacterium and defense response to Gram-positive bacterium. Predicted to localize to several cellular components, including Golgi cis cisterna; cytoplasmic vesicle; and rough endoplasmic reticulum lumen. Is expressed in several structures, including forelimb bud; liver; mesothelium; small intestine; and yolk sac |
| **Hist1h2ao; Hist1h2ap** | histone cluster 1, H2ao; histone cluster 1, H2ap | -1.98 | 0.0013 | Predicted to have DNA binding activity. Predicted to be involved in chromatin silencing and negative regulation of cell population proliferation. Predicted to localize to nuclear chromatin. |
| **Ctsg** | cathepsin G | -1.93 | 0.0008 | Predicted to have heparin binding activity and serine-type endopeptidase activity. Involved in defense response to fungus; positive regulation of immune response; and response to bacterium. Predicted to localize to several cellular components, including cytoplasmic stress granule; plasma membrane; and secretory granule. Is expressed in liver. |
| **Gpaa1** | GPI anchor attachment protein 1 | -1.45 | 0.0127 | Predicted to contribute to GPI anchor binding activity, to be involved in attachment of GPI anchor to protein and protein-containing complex assembly and, to localize to GPI-anchor transamidase complex and integral component of plasma membrane. |
| **Crisp2** | cysteine-rich secretory protein 2 | -1.45 | 0.0153 | Predicted to be involved in cell-cell adhesion, to localize to extracellular space. |
| **Lcn2** | lipocalin 2 | -1.44 | 0.0002 | Exhibits iron ion binding activity. Involved in several processes, including extrinsic apoptotic signaling pathway in absence of ligand; positive regulation of cold-induced thermogenesis; and siderophore transport. It is expressed in several structures, including genitourinary system; heart; liver; lung; and skeletal muscle. |
| **Prmt3** | protein arginine N-methyltransferase 3 | -1.44 | 0.0164 | Exhibits protein-arginine N-methyltransferase activity and ribosome binding activity. Involved in protein methylation. Localizes to cytosol. Is expressed in several structures, including central nervous system; genitourinary system; liver; sensory organ; and spleen. |
| **Nfe2l3** | nuclear factor, erythroid derived 2, like 3 | -1.42 | 0.0133 | Exhibits DNA-binding transcription repressor activity, RNA polymerase II-specific and RNA polymerase II cis-regulatory region sequence-specific DNA binding activity. Involved in negative regulation of transcription by RNA polymerase II. Predicted to localize to cytoplasm and nucleus. Is expressed in brain; forelimb bud; gonad; and ureter. |
| **Map6** | microtubule-associated protein 6 | -1.42 | 0.0152 | Exhibits microtubule binding activity. Predicted to be involved in several processes, including axonal transport of mitochondrion; generation of neurons; and lysosome localization. Predicted to localize to several cellular components, including cis-Golgi network; cytoplasmic vesicle; and perinuclear region of cytoplasm. It is expressed in cranial ganglion; dorsal root ganglion; and sensory organ. |

*Gene function as reported in NCBI ( https://www.ncbi.nlm.nih.gov/guide/howto/find-func-gene/ )

## Supplementary table 8. Top significantly enriched KEGG signaling pathways in NSCs of normal mice treated with luteolin (PBS+L) compared to untreated normal mice (PBS)

| Regulation | KEGG signaling | Genes |
|---|---|---|
| Upregulation | **Wnt signaling pathway (mmu04310)** | adenomatosis polyposis coli 2(***Apc2***)<br>calcium/calmodulin-dependent protein kinase II alpha(***Camk2a***)<br>cyclin D2(***Ccnd2***)<br>prickle planar cell polarity protein 2(***Prickle2***)<br>transcription factor 7 like 1 (T cell specific, HMG box)(***Tcf7l1***)<br>wingless-type MMTV integration site family, member 11(***Wnt11***) |
| Downregulation | **Parkinson's disease (mmu05012)** | ATP synthase, H+ transporting, mitochondrial F0 complex, subunit C3 (subunit 9)(***Atp5g3***)<br>ATP synthase, H+ transporting, mitochondrial F0 complex, subunit D(***Atp5h***)<br>ATP synthase, H+ transporting, mitochondrial F0 complex, subunit F(***Atp5j***)<br>NADH dehydrogenase (ubiquinone) 1 alpha subcomplex, 13(***Ndufa13***)<br>NADH dehydrogenase (ubiquinone) 1 beta subcomplex, 11(***Ndufb11***)<br>NADH dehydrogenase subunit 5(***ND5***)<br>cytochrome c oxidase subunit IV isoform 1(***Cox4i1***)<br>Cytochrome c oxidase subunit VIa polypeptide 1(***Cox6a1***)<br>cytochrome c oxidase subunit Vb(***Cox5b***)<br>guanine nucleotide binding protein (G protein), alpha inhibiting 2(***Gnai2***)<br>solute carrier family 25 (mitochondrial carrier, adenine nucleotide translocator), member 5(***Slc25a5***)<br>Succinate dehydrogenase complex, subunit A, flavoprotein (Fp)(***Sdha***)<br>ubiquinol-cytochrome c reductase binding protein (***Uqcrb***) |
| | **Alzheimer's disease (mmu05010)** | ATP synthase, H+ transporting, mitochondrial F0 complex, subunit C3 (subunit 9)(***Atp5g3***)<br>ATP synthase, H+ transporting, mitochondrial F0 complex, subunit D(***Atp5h***)<br>ATP synthase, H+ transporting, mitochondrial F0 complex, subunit F(***Atp5j***)<br>NADH dehydrogenase (ubiquinone) 1 alpha subcomplex, 13(***Ndufa13)***<br>NADH dehydrogenase (ubiquinone) 1 beta subcomplex, 11(***Ndufb11***)<br>amyloid beta (A4) precursor protein (***App***)<br>apolipoprotein E(***Apoe***)<br>calmodulin 1(***Calm1***)<br>cytochrome c oxidase subunit IV isoform 1(***Cox4i1***)<br>cytochrome c oxidase subunit VIa polypeptide 1(***Cox6a1***)<br>cytochrome c oxidase subunit Vb(***Cox5b***)<br>glyceraldehyde-3-phosphate dehydrogenase, pseudogene 15(***Gapdh-ps15***)<br>Succinate dehydrogenase complex, subunit A, flavoprotein (Fp)(***Sdha***)<br>ubiquinol-cytochrome c reductase binding protein (***Uqcrb***) |
| | **Dopaminergic synapse (mmu04728)** | FBJ osteosarcoma oncogene (***Fos***)<br>GNAS (guanine nucleotide binding protein, alpha stimulating) complex locus (***Gnas***)<br>cAMP responsive element binding protein 3(***Creb3***)<br>calcium/calmodulin-dependent protein kinase II alpha (***Camk2a***)<br>calmodulin 1(***Calm1***)<br>guanine nucleotide binding protein (G protein), alpha inhibiting 2(***Gnai2***)<br>guanine nucleotide binding protein (G protein), beta 1(***Gnb1***)<br>guanine nucleotide binding protein (G protein), gamma 11(***Gng11***)<br>predicted gene 15776(***Gm15776***)<br>protein phosphatase 2, regulatory subunit B, alpha (***Ppp2r2a***) |
| | **HIF-1 signaling pathway (mmu04066)** | FMS-like tyrosine kinase 1(***Flt1***)<br>calcium/calmodulin-dependent protein kinase II alpha (***Camk2a***)<br>eukaryotic translation initiation factor 4E member 2(***Eif4e2***)<br>glyceraldehyde-3-phosphate dehydrogenase, pseudogene 15(***Gapdh-ps15***)<br>predicted gene 9840(***Gm9840***)<br>ribosomal protein S6(***Rps6***)<br>solute carrier family 2 (facilitated glucose transporter), member 1(***Slc2a1***)<br>transferrin receptor (***Tfrc***) |
| | **Gap junction (mmu04540)** | GNAS (guanine nucleotide binding protein, alpha stimulating) complex locus (***Gnas***)<br>gap junction protein, alpha 1(***Gja1***)<br>gap junction protein, delta 2(***Gjd2***)<br>guanine nucleotide binding protein (G protein), alpha inhibiting 2(***Gnai2***)<br>tubulin, alpha 1A(***Tuba1a***)<br>tubulin, alpha 1B(***Tuba1b***)<br>tubulin, beta 4B class IVB(***Tubb4b***) |
| | **Thyroid hormone signaling pathway (mmu04919)** | ATPase, Na+/K+ transporting, alpha 2 polypeptide (***Atp1a2***)<br>ATPase, Na+/K+ transporting, beta 2 polypeptide (***Atp1b2***)<br>actin, beta (***Actb***)<br>actin, gamma, cytoplasmic 1(***Actg1***)<br>catenin (cadherin associated protein), beta 1(***Ctnnb1***)<br>notch 1(***Notch1***)<br>solute carrier family 2 (facilitated glucose transporter), member 1(***Slc2a1***)<br>solute carrier organic anion transporter family, member 1c1(***Slco1c1***) |
| | **Tight junction (mmu04530)** | actin, beta (***Actb***)<br>actin, gamma, cytoplasmic 1(***Actg1***)<br>claudin 5(***Cldn5***)<br>myosin, light chain 12A, regulatory, non-sarcomeric (***Myl12a***)<br>myosin, light chain 12B, regulatory (***Myl12b***)<br>protein phosphatase 2, regulatory subunit B, alpha (***Ppp2r2a***)<br>tight junction associated protein 1(***Tjap1***) |
| | **Hippo signaling pathway (mmu04390)** | Ras association (RalGDS/AF-6) domain family member 1(***Rassf1***)<br>actin, beta (***Actb***)<br>actin, gamma, cytoplasmic 1(***Actg1***)<br>catenin (cadherin associated protein), beta 1(***Ctnnb1***)<br>inhibitor of DNA binding 1(***Id1***)<br>large tumor suppressor 2(***Lats2***)<br>protein phosphatase 2, regulatory subunit B, alpha (***Ppp2r2a***)<br>tyrosine 3-monooxygenase/tryptophan 5-monooxygenase activation protein, epsilon polypeptide (***Ywhae***)<br>tyrosine 3-monooxygenase/tryptophan 5-monooxygenase activation protein, theta polypeptide (***Ywhaq***) |

**Supplementary table 9. Top 10 upregulated genes in the hippocampus of LPS-induced depression mice treated with luteolin (LPS+L) compared to untreated LPS-induced depression mice (LPS).**

| Gene symbol | Gene title | Fold change | *p*-value | Gene function* |
|---|---|---|---|---|
| **Fmod** | fibromodulin | -5.05 | 0.0053 | Encodes fibromodulin protein belonging to the family of small interstitial proteoglycans. Owing to the interaction with type I and type II collagen fibrils and in vitro inhibition of fibrillogenesis, the encoded protein may play a role in the assembly of extracellular matrix. It may also regulate TGF-beta activities by sequestering TGF-beta into the extracellular matrix. |
| **Ttr** | transthyretin | -3.61 | 0.0061 | This gene encodes a carrier protein responsible for the transport of thyroid hormones and retinol. |
| **Pigz** | phosphatidylinositol glycan anchor biosynthesis, class Z | -3.38 | 5.85E-06 | The glycosylphosphatidylinositol (GPI) anchor is a glycolipid found on many blood cells that serves to anchor proteins to the cell surface. |
| **Xlr3a; Xlr3b; Xlr3c** | X-linked lymphocyte-regulated 3A; X-linked lymphocyte-regulated 3B; X-linked lymphocyte-regulated 3C | -3.18 | 0.0005 | This gene is predicted to be involved in meiotic cell cycle and spermatid development. |
| **Gfap** | glial fibrillary acidic protein | -3.11 | 3.01E-05 | Encodes the GFAP protein, a structural constituent of cytoskeleton. Involved in several processes, including long-term synaptic potentiation; and neurogenesis. Localizes to several cellular components, including astrocyte end-foot; cell body; and intermediate filament. |
| **Kbtbd11** | kelch repeat and BTB (POZ) domain containing 11 | -3.07 | 0.0011 | Is expressed in several structures, including central nervous system; early tubule; ganglia; lung; and sensory organ. |
| **Tmsb15b2; Tmsb15l** | thymosin beta 15b2; thymosin beta 15b like | -2.95 | 0.0044 | Exhibits actin monomer binding activity. Involved in negative regulation of stress fiber assembly and sequestering of actin monomers. Localizes to filamentous actin and stress fiber. |
| **Ptgds** | prostaglandin D2 synthase (brain) | -2.89 | 1.01E-05 | It is a protein coding gene. Exhibits prostaglandin-D synthase activity and retinoid binding activity. Involved in prostaglandin biosynthetic process; and regulation of circadian sleep/wake cycle, sleep. Localizes to extracellular region. Is expressed in several structures, including nervous system; and sensory organ. |
| **Vwf** | Von Willebrand factor homolog | -2.84 | 0.0213 | It is a protein coding gene. Predicted to have several functions, including chaperone binding activity; identical protein binding activity; and integrin binding activity. Involved in animal organ development; cell-substrate adhesion; and platelet activation. Is expressed in several structures, including leptomeninges. |
| **Adat2** | adenosine deaminase, tRNA-specific 2 | -2.79 | 7.44E-08 | It is a protein coding gene. Predicted to have tRNA-specific adenosine-34 deaminase activity. Predicted to be involved in tRNA wobble adenosine to inosine editing. Predicted to localize to tRNA-specific adenosine-34 deaminase complex. |

*Gene function as reported in NCBI ( https://www.ncbi.nlm.nih.gov/guide/howto/find-func-gene/ )

**Supplementary table 10. Top 10 downregulated genes in the hippocampus of LPS-induced depression mice treated with luteolin compared to untreated LPS-induced depression mice.**

| Gene symbol | Gene title | Fold change | *p*-value | Gene function* |
|---|---|---|---|---|
| **Phgdh** | 3-phosphoglycerate dehydrogenase | 7.29 | 0.0018 | It is a protein coding gene, predicted to have phosphoglycerate dehydrogenase activity. Involved in several processes, including G1 to G0 transition; cellular amino acid metabolic process; and nervous system development. Localizes to myelin sheath. Is expressed in several structures, including; brain; and sensory organ. |
| **Zc2hc1a** | zinc finger, C2HC-type containing 1A | 4.22 | 1.23E-16 | It is a protein coding gene, predicted to have metal ion binding activity. Is expressed in central nervous system; dorsal root ganglion; early conceptus; olfactory epithelium; |
| **Rbm45** | RNA binding motif protein 45 | 2.99 | 1.24E-06 | It is a protein coding gene, predicted to have RNA binding activity and identical protein binding activity. Predicted to be involved in cell differentiation and nervous system development. Is expressed in brain; sensory organ; and spinal cord. |
| **Scd2** | stearoyl-Coenzyme A desaturase 2 | 2.94 | 0.0335 | Exhibits palmitoyl-CoA 9-desaturase activity and stearoyl-CoA 9-desaturase activity. Involved in monounsaturated fatty acid biosynthetic process. Localizes to endoplasmic reticulum. Is expressed in several structures, including central nervous system, and sensory organ. |
| **Camk2n1** | calcium/calmodulin-dependent protein kinase II inhibitor 1 | 2.98 | 5.46E-08 | Exhibits protein kinase inhibitor activity. Predicted to be involved in several processes, including negative regulation of cellular protein metabolic process; positive regulation of insulin secretion involved in cellular response to glucose stimulus; and positive regulation of systemic arterial blood pressure. Localizes to postsynaptic density. Is expressed in several structures, including brain. |
| **Tgtp1; Tgtp2** | T cell specific GTPase 1; T cell specific GTPase 2 | 1.98 | 0.0007 | Predicted to be involved in cellular response to interferon-beta and defense response. Predicted to localize to endoplasmic reticulum membrane. Is expressed in several structures, including genitourinary system; hemolymphoid system; integumental system; nervous system; and nose. |
| **Pisd-ps1** | phosphatidylserine decarboxylase, pseudogene 1 | 1.99 | 0.0127 | It is a pseudogene, expressed in nervous system and olfactory epithelium. |
| **Pisd-ps3** | phosphatidylserine decarboxylase, pseudogene 3 | 2.16 | 0.0117 | It is a pseudogene, expressed in nervous system and olfactory epithelium. |
| **Erdr1** | erythroid differentiation regulator 1 | 2.26 | 6.01E-10 | It is a protein coding gene involved in negative regulation of cell migration; negative regulation of cell population proliferation; and somatic stem cell population maintenance. It is expressed in several structures, including central nervous system and retina. |
| **H2-K1** | histocompatibility 2, K1, K region | 1.96 | 6.90E-09 | Exhibits MHC class I protein binding activity and peptide antigen binding activity. Involved in several processes, including antigen processing and presentation of endogenous peptide antigen via MHC class I via ER pathway, TAP-dependent; antigen processing and presentation of exogenous peptide antigen via MHC class I; and positive regulation of T cell mediated cytotoxicity. Localizes to external side of plasma membrane. Is expressed in several structures, including brain. |

*Gene function as reported in NCBI ( https://www.ncbi.nlm.nih.gov/guide/howto/find-func-gene/ )

**Supplementary table 11. Top significantly enriched KEGG signaling pathways in hippocampus of normal mice treated with luteolin (PBS+L) compared to untreated normal mice (PBS)**

| Regulation | KEGG signaling | Genes |
|---|---|---|
| Upregulation | **Dopaminergic synapse (mmu04728)** | activating transcription factor 2(***Atf2***)<br>cAMP responsive element binding protein 3(***Creb3***)<br>guanine nucleotide binding protein (G protein), alpha inhibiting 1(***Gnai1***)<br>guanine nucleotide binding protein (G protein), gamma 12(***Gng12***)<br>guanine nucleotide binding protein (G protein), gamma 4(***Gng4***)<br>kinesin family member 5A(***Kif5a***)<br>protein kinase, cAMP dependent, catalytic, beta (***Prkacb***)<br>protein phosphatase 2 (formerly 2A), catalytic subunit, alpha isoform (***Ppp2ca***)<br>protein phosphatase 2 (formerly 2A), catalytic subunit, beta isoform (***Ppp2cb***) |
| | **GABAergic synapse (mmu04727)** | adenylate cyclase 8(***Adcy8***)<br>gamma-aminobutyric acid (GABA) A receptor, subunit beta 1(***Gabrb1***)<br>glutaminase (***Gls***)<br>guanine nucleotide binding protein (G protein), alpha inhibiting 1(***Gnai1***)<br>guanine nucleotide binding protein (G protein), gamma 12(***Gng12***)<br>guanine nucleotide binding protein (G protein), gamma 4(***Gng4***)<br>protein kinase, cAMP dependent, catalytic, beta (***Prkacb***) |
| | **Long term potentiation (mmu04720)** | Braf transforming gene (***Braf)***<br>Kirsten rat sarcoma viral oncogene homolog (***Kras***)<br>adenylate cyclase 8(***Adcy8***)<br>glutamate receptor, metabotropic 5(***Grm5***)<br>protein kinase, cAMP dependent, catalytic, beta (***Prkacb***)<br>ribosomal protein S6 kinase polypeptide 6(***Rps6ka6***) |
| | **Synaptic vesicle cycle (mmu04721)** | ATPase, H+ transporting, lysosomal V0 subunit E2(***Atp6v0e2***)<br>ATPase, H+ transporting, lysosomal V1 subunit B2(***Atp6v1b2***)<br>adaptor-related protein complex 2, alpha 2 subunit (***Ap2a2***)<br>synaptotagmin I(***Syt1***)<br>syntaxin 1B(***Stx1b***)<br>syntaxin 3(***Stx3***) |
| | **Neurotrophin signaling pathway (mmu04722)** | Braf transforming gene (***Braf***)<br>Kirsten rat sarcoma viral oncogene homolog (***Kras***)<br>kinase D-interacting substrate 220 (***Kidins220***)<br>neurotrophic tyrosine kinase, receptor, type 2(***Ntrk2***)<br>neurotrophic tyrosine kinase, receptor, type 3(***Ntrk3***)<br>phosphatidylinositol 3-kinase, catalytic, alpha polypeptide (***Pik3ca***)<br>ribosomal protein S6 kinase polypeptide 6(***Rps6ka6***)<br>ribosomal protein S6 kinase, polypeptide 5(***Rps6ka5***)<br>tyrosine 3-monooxygenase/tryptophan 5-monooxygenase activation protein, epsilon polypeptide (***Ywhae***) |
| | **Glutamatergic synapse (mmu04724)** | adenylate cyclase 8(***Adcy8***)<br>glutamate receptor ionotropic, NMDA3A(***Grin3a***)<br>glutamate receptor, metabotropic 5(***Grm5***)<br>glutamate receptor, metabotropic 7(***Grm7***)<br>glutaminase (***Gls***)<br>guanine nucleotide binding protein (G protein), alpha inhibiting 1(***Gnai1***)<br>guanine nucleotide binding protein (G protein), gamma 12(***Gng12***)<br>guanine nucleotide binding protein (G protein), gamma 4(***Gng4***)<br>protein kinase, cAMP dependent, catalytic, beta (***Prkacb***) |
| | **Gap junction (mmu04540)** | Kirsten rat sarcoma viral oncogene homolog (***Kras***)<br>adenylate cyclase 8(***Adcy8***)<br>glutamate receptor, metabotropic 5 (***Grm5***)<br>guanine nucleotide binding protein (G protein), alpha inhibiting 1(***Gnai1***)<br>guanylate cyclase 1, soluble, alpha 2(***Gucy1a2***)<br>guanylate cyclase 1, soluble, beta 3(***Gucy1b3***)<br>platelet derived growth factor, B polypeptide (***Pdgfb***)<br>protein kinase, cAMP dependent, catalytic, beta (***Prkacb***) |
| | **Hippo signaling pathway (mmu04390)** | bone morphogenetic protein receptor, type 1A(***Bmpr1a***)<br>bone morphogenetic protein receptor, type II (serine/threonine kinase) (***Bmpr2***)<br>catenin (cadherin associated protein), beta 1(***Ctnnb1***)<br>discs, large homolog 2 (Drosophila)(***Dlg2***)<br>frizzled class receptor 3(***Fzd3***)<br>neurofibromatosis 2(***Nf2***)<br>protein phosphatase 2 (formerly 2A), catalytic subunit, alpha isoform (***Ppp2ca***)<br>protein phosphatase 2 (formerly 2A), catalytic subunit, beta isoform (***Ppp2cb***)<br>tyrosine 3-monooxygenase/tryptophan 5-monooxygenase activation protein, epsilon polypeptide (***Ywha***e)<br>wingless-type MMTV integration site family, member 10A(***Wnt10a***)<br>wingless-type MMTV integration site family, member 5A(***Wnt5a***)<br>wingless-type MMTV integration site family, member 7B(***Wnt7b***) |
| | **Axon guidance (mmu04360)** | Eph receptor A3(***Epha3***)<br>Kirsten rat sarcoma viral oncogene homolog (***Kras)***<br>deleted in colorectal carcinoma ***(Dcc)***<br>ephrin B2(***Efnb2***)<br>guanine nucleotide binding protein (G protein), alpha inhibiting 1(***Gnai1***)<br>met proto-oncogene (***Met***)<br>p21 protein (Cdc42/Rac)-activated kinase 1(***Pak1***)<br>p21 protein (Cdc42/Rac)-activated kinase 3(***Pak3***)<br>plexin C1(***Plxnc1***)<br>slit homolog 2 (Drosophila)(***Slit2***)<br>unc-5 netrin receptor D (***Unc5d***) |

**Supplementary table 11. (continued)**

| Regulation | KEGG signaling | Genes |
|---|---|---|
| Upregulation | **Ras signaling pathway (mmu04014)** | Kirsten rat sarcoma viral oncogene homolog (***Kras***)<br>RAS p21 protein activator 2(***Rasa2***)<br>Ras association (RalGDS/AF-6) domain family member 5(***Rassf5***)<br>fibroblast growth factor 12(***Fgf12***)<br>fibroblast growth factor receptor 3(***Fgfr3***)<br>guanine nucleotide binding protein (G protein), gamma 12(***Gng12***)<br>guanine nucleotide binding protein (G protein), gamma 4(***Gng4***)<br>kit ligand (***Kitl***)<br>met proto-oncogene (***Met***)<br>p21 protein (Cdc42/Rac)-activated kinase 1(***Pak1***)<br>p21 protein (Cdc42/Rac)-activated kinase 3(***Pak3***)<br>phosphatidylinositol 3-kinase, catalytic, alpha polypeptide (***Pik3ca***)<br>platelet derived growth factor, B polypeptide (***Pdgfb***)<br>protein kinase, cAMP dependent, catalytic, beta (***Prkacb***)<br>v-ral simian leukemia viral oncogene A (ras related) (***Rala***)<br>vascular endothelial growth factor D(***Vegfd***) |
| | **PI3K-Akt signaling pathway (mmu04151)** | Kirsten rat sarcoma viral oncogene homolog (***Kras***)<br>activating transcription factor 2(***Atf2***)<br>cAMP responsive element binding protein 3(***Creb3)***<br>collagen, type I, alpha 1(***Col1a1***)<br>fibroblast growth factor 12(***Fgf12***)<br>fibroblast growth factor receptor 3(***Fgfr3***)<br>guanine nucleotide binding protein (G protein), gamma 12(***Gng12***)<br>guanine nucleotide binding protein (G protein), gamma 4(***Gng4***)<br>integrin alpha 4(***Itga4***)<br>interferon (alpha and beta) receptor 1(***Ifnar1***)<br>kit ligand (***Kitl***)<br>met proto-oncogene (***Met***)<br>phosphatase and tensin homolog (***Pten***)<br>phosphatidylinositol 3-kinase, catalytic, alpha polypeptide (***Pik3ca***)<br>platelet derived growth factor, B polypeptide (***Pdgfb***)<br>protein kinase, AMP-activated, alpha 2 catalytic subunit (***Prkaa2***)<br>protein phosphatase 2 (formerly 2A), catalytic subunit, alpha isoform (***Ppp2ca***)<br>protein phosphatase 2 (formerly 2A), catalytic subunit, beta isoform (***Ppp2cb***)<br>ribosomal protein S6(***Rps6***)<br>tyrosine 3-monooxygenase/tryptophan 5-monooxygenase activation protein, epsilon polypeptide (***Ywhae***)<br>vascular endothelial growth factor D(***Vegfd***)<br>vitronectin (***Vtn***) |
| | **Signaling pathways regulating pluripotency of stem cells (mmu04550)** | Kirsten rat sarcoma viral oncogene homolog (***Kras***)<br>SKI-like (***Skil***)<br>activin receptor IIA(***Acvr2a***)<br>bone morphogenetic protein receptor, type 1A(***Bmpr1a***)<br>bone morphogenetic protein receptor, type II (serine/threonine kinase) (***Bmpr2***)<br>catenin (cadherin associated protein), beta 1(***Ctnnb1***)<br>fibroblast growth factor receptor 3(***Fgfr3***)<br>frizzled class receptor 3(***Fzd3***)<br>phosphatidylinositol 3-kinase, catalytic, alpha polypeptide (***Pik3ca***)<br>wingless-type MMTV integration site family, member 10A(***Wnt10a***)<br>wingless-type MMTV integration site family, member 5A (***Wnt5a***)<br>wingless-type MMTV integration site family, member 7B (***Wnt7b***) |
| | **Cholinergic synapse (mmu04725)** | Kirsten rat sarcoma viral oncogene homolog (***Kras***)<br>adenylate cyclase 8(***Adcy8***)<br>cAMP responsive element binding protein 3(***Creb3***)<br>cholinergic receptor, nicotinic, alpha polypeptide 4(***Chrna4***)<br>guanine nucleotide binding protein (G protein), alpha inhibiting 1(***Gnai1***)<br>guanine nucleotide binding protein (G protein), gamma 12(***Gng12***)<br>guanine nucleotide binding protein (G protein), gamma 4(***Gng4***)<br>phosphatidylinositol 3-kinase, catalytic, alpha polypeptide (***Pik3ca***)<br>potassium voltage-gated channel, subfamily Q, member 2(***Kcnq2***)<br>potassium voltage-gated channel, subfamily Q, member 5(***Kcnq5***)<br>protein kinase, cAMP dependent, catalytic, beta (***Prkacb***) |
| | **MAPK signaling pathway (mmu04010)** | Braf transforming gene (***Braf***)<br>Kirsten rat sarcoma viral oncogene homolog (***Kras***)<br>RAS p21 protein activator 2(***Rasa2***)<br>activating transcription factor 2(***Atf2***)<br>calcium channel, voltage-dependent, alpha2/delta subunit 1(***Cacna2d1***)<br>calcium channel, voltage-dependent, gamma subunit 3(***Cacng3***)<br>fibroblast growth factor 12(***Fgf12***)<br>fibroblast growth factor receptor 3(***Fgfr3***)<br>guanine nucleotide binding protein (G protein), gamma 12(***Gng12***)<br>Microtubule-associated protein tau (***Mapt***)<br>mitogen-activated protein kinase 8 interacting protein 3(***Mapk8ip3***)<br>mitogen-activated protein kinase kinase 4(***Map2k4***)<br>neurotrophic tyrosine kinase, receptor, type 2(***Ntrk2***)<br>p21 protein (Cdc42/Rac)-activated kinase 1(***Pak1***)<br>platelet derived growth factor, B polypeptide (***Pdgfb***)<br>protein kinase, cAMP dependent, catalytic, beta (***Prkacb***)<br>protein phosphatase 1A, magnesium dependent, alpha isoform (***Ppm1a***)<br>ribosomal protein S6 kinase polypeptide 6(***Rps6ka6***)<br>ribosomal protein S6 kinase, polypeptide 5(***Rps6ka5***) |
